# CATBOSS: Cluster Analysis of Trajectories Based on Segment Splitting

**DOI:** 10.1021/acs.jcim.1c00598

**Published:** 2021-10-05

**Authors:** Jovan Damjanovic, James M. Murphy, Yu-Shan Lin

**Affiliations:** † Department of Chemistry, Tufts University, Medford, Massachusetts 02155, United States; ‡ Department of Mathematics, Tufts University, Medford, Massachusetts 02155, United States

## Abstract

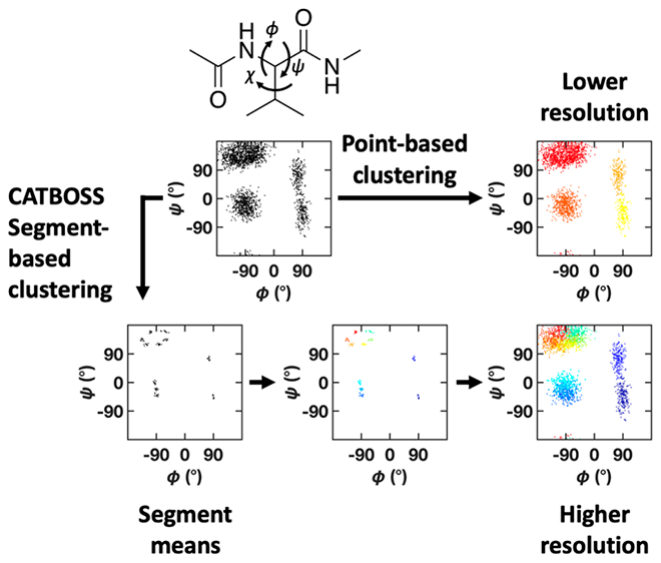

Molecular dynamics (MD) simulations are an exceedingly and increasingly potent tool for molecular behavior prediction and analysis. However, the enormous wealth of data generated by these simulations can be difficult to process and render in a human-readable fashion. Cluster analysis is a commonly used way to partition data into structurally distinct states. We present a method that improves on the state of the art by taking advantage of the temporal information of MD trajectories to enable more accurate clustering at a lower memory cost. To date, cluster analysis of MD simulations has generally treated simulation snapshots as a mere collection of independent data points and attempted to separate them into different clusters based on structural similarity. This new method, cluster analysis of trajectories based on segment splitting (CATBOSS), applies density-peak-based clustering to classify *trajectory segments* learned by change detection. Applying the method to a synthetic toy model as well as four real-life data sets–trajectories of MD simulations of alanine dipeptide and valine dipeptide as well as two fast-folding proteins–we find CATBOSS to be robust and highly performant, yielding natural-looking cluster boundaries and greatly improving clustering resolution. As the classification of points into segments emphasizes density gaps in the data by grouping them close to the state means, CATBOSS applied to the valine dipeptide system is even able to account for a degree of freedom deliberately omitted from the input data set. We also demonstrate the potential utility of CATBOSS in distinguishing metastable states from transition segments as well as promising application to cases where there is little or no advance knowledge of intrinsic coordinates, making for a highly versatile analysis tool.

## 1 Introduction

With recent developments in both high-performance computing hardware and simulation algorithms, molecular dynamics (MD) simulations have risen from a predominantly explanatory technique to an invaluable tool for molecular behavior prediction.^1−4^ Fast network interconnect protocols,^5,6^ GPU-based acceleration,^7−10^ and architecture-specific algorithms^11^ have allowed scientists to probe micro- and even millisecond time scales as well as systems with thousands of atoms.^12^ A natural consequence of these developments is the enormous amount of data generated, necessitating robust analysis methods.^13,14^ As part of effective data processing, cluster analysis is frequently employed to partition structurally similar data points into states.

Early landmark efforts in the field of cluster analysis include approaches such as *k*-means and *k*-medoids, which aim to minimize the distance between data points and points identified as cluster centroids.^15−18^ The primary limitations of such methods include difficulty handling clusters that are not highly spherical (this issue may be addressed using kernel *k*-means or spectral clustering),^19,20^ as well as the need for the user to *a priori* specify the number of centroids, *k*. The time complexity of these algorithms in their native form is *O*(*n*^2^), where *n* is the number of data points, with further refinements proposed.^21,22^ An alternative commonly used approach, agglomerative hierarchical clustering, instead yields a family of clustering schemes, starting from all points in separate states and gradually merging points based on a distance metric until all points are in the same state, wherein the user specifies the number of clusters by cutting a dendrogram upon completion of the algorithm.^23,24^ This approach has a worst-case cubic time complexity,^25^ with *O*(*n*^2^) achieved by optimized variants.^26^

In recent years, a density-peak-based approach proposed by Rodriguez and Laio has established itself as the state of the art.^27^ This method relies on the observation that cluster centroids exhibit a relatively high local density compared to their neighbors and a large distance from any points of higher density. This method has proven competent at handling clusters of varying shapes, sizes, and densities and has already been applied effectively to MD data sets.^28−34^ Limitations of this method previously identified by the scientific community include the need for the user to specify the cutoff distance for the kernel density estimator and the need for the user to visually inspect the generated decision graph and manually assign cluster centroids as well as quadratic memory complexity.^35,36^ The last issue in particular can make memory requirements for a typical MD data set balloon to hundreds of gigabytes, necessitating the use of expensive high-end hardware. This problem can be mitigated by recomputing the pairwise distances as needed, rather than storing them (which trades memory for computational complexity) or using local approximations for density estimation.^36^ Later implementations of the method may be run on large data sets on regular desktop machines.^37^ Several other groups have also proposed extensions of the method that address the aforementioned shortcomings;^35,36^ however, none have, to our knowledge, entirely eliminated user input or reduced memory complexity without computational trade-off or the use of approximations.

**Figure 1:**
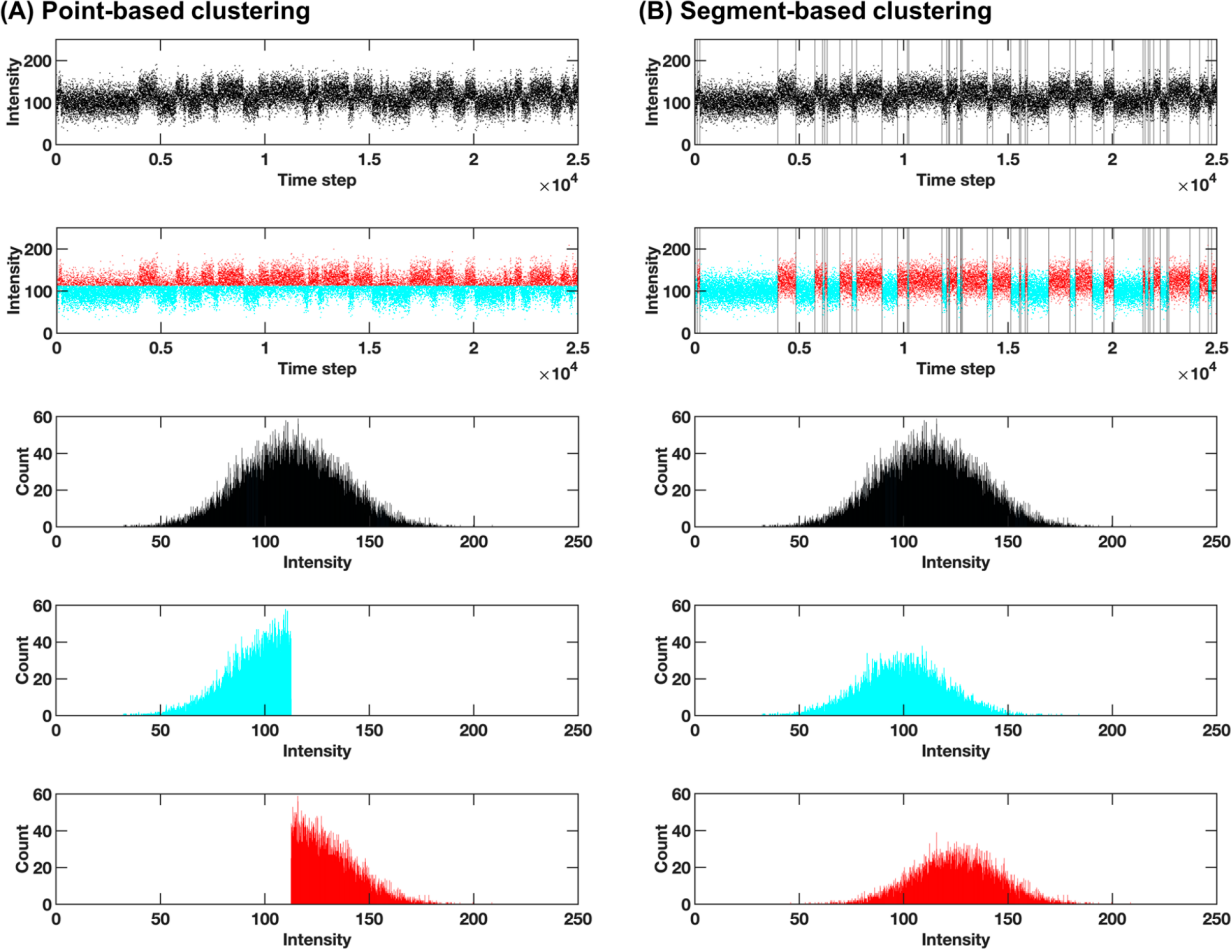
Example two-state synthetic trajectory clustered (A) by data points and (B) by segments. Each panel shows from top to bottom: the raw trajectory (in the case of segment-based clustering, with change points indicated by vertical lines); the trajectory labeled by state assignments following clustering; a histogram representing the (apparent unimodal) data distribution; and histograms representing the data distribution in each of the two clusters learned by the two approaches. Note the sharp boundary between states in the point-based case and distribution symmetry in the segment-based case; the latter suggesting data sampled from a distribution oscillating around a given mean (consistent with the ground truth).

In contrast to clustering a data set purely based on structural similarity among data points, clustering the data set based on segments can enable users to obtain a better and more natural picture of the metastable states (Figure 1). Segment-based clustering of time series data has been previously applied to short, low-dimensional time series, such as single-molecule spectroscopy data.^38^ Such clustering is accomplished by applying a change detection method to the time series to identify the segments and partitioning them into clusters based on a given dissimilarity measure. A 2019 publication by Li and Yang demonstrated a high level of robustness and accuracy on one-dimensional (1D), two-state synthetic data.^38^ The ability of this method to account for overlap in data distributions represents a notable advance in the handling of time series data. However, this method performs change detection by recursive likelihood estimation—in each iteration, the most likely change point is determined, and its likelihood is compared to a tunable critical value. Once a change point is established, the trajectory is split into two fragments at that point, and the procedure is applied recursively to the resulting fragments.^37^ The complexity of the recursive change detection becomes a computational bottleneck when applied to large, multidimensional MD data sets.

In this work, we present a method similar in spirit, dubbed cluster analysis of trajectories based on segment splitting (CATBOSS), which uses density-peak clustering to cluster trajectory segments, rather than data points. To our knowledge, CATBOSS is the first segment-based clustering protocol effectively applied to MD trajectory data. As part of CATBOSS, trajectory segments are demarcated using SIMPLE, a change detection algorithm developed by Fan et al.^39^ This particular change detection method was chosen for its ability to recognize correlated changes, which are frequently present in MD data. In contrast to Euclidean distance between pairs of data points, we use the earth mover’s distance metric to determine the distance between trajectory segments. Earth mover’s distance naturally extends the idea of distance between points to that between collections of points, is perceptually meaningful, and is a true metric for a metric ground distance.^40^ Distances between segments are then passed to density-peak clustering, with each segment’s local density contribution scaled linearly by its length. The resulting density profile, along with tight grouping of segment means in the vicinity of state centers in data coordinate space, makes for prominent, easily resolved density peaks, which motivated the choice of clustering algorithm. Applying CATBOSS to a batch toy model similar to the 1D, two-state synthetic data used by Li and Yang,^38^ we show that compared to the previously developed method, CATBOSS maintains an extremely high accuracy even when the two states are sampled from highly overlapping distributions, or when one state has a much greater population than the other. Moreover, using a pair of real-world MD data sets—alanine dipeptide and valine dipeptide—we demonstrate that CATBOSS yields a natural partitioning of the Ramachandran plot while dramatically lowering the number of pairwise distances used for the clustering. Further, on the example of valine dipeptide, we demonstrate an increase in resolution which allows our method to identify clusters corresponding to different side chain rotamers even when given only backbone dihedral values. By analyzing the slope and length distribution of trajectory segments, we show the ability of our method to distinguish metastable states and transition segments, providing valuable information about the dynamics, in addition to the structure of the simulated systems. As testing on simple model systems has yielded highly promising results, we have also applied CATBOSS to two MD trajectories of fast-folding protein systems that have previously been used for clustering algorithm validation. We report the partitioning results for the trajectory of folded dynamics of bovine pancreatic trypsin inhibitor (BPTI),^12^ in comparison to another recently published method, Sapphire-based clustering.^41^ In addition, we also consider the CATBOSS partitioning of a long (∼1.5 × 10^6^ frames) trajectory of the Nle/Nle mutant of the villin headpiece 35-residue subdomain (HP35) at 360 K,^42^ in comparison to most probable path clustering by Jain and Stock.^43^ This trajectory also serves as further proof of scaling of the method presented here, where we demonstrate the ability to handle a data set with a high number of entries as well as a high-dimensional data set. We apply our method to time series of both low-dimensional intrinsic coordinates as well as all relevant backbone dihedral angles. In all of these cases, we find that CATBOSS matches or outperforms the previously reported methods. Lastly, we show that storing intersegment (as opposed to interpoint) distances results in an orders-of-magnitude decrease in memory complexity compared to the base density-peak implementation which takes a distance matrix as input, making CATBOSS a versatile choice for a wide array of systems.

One caveat that bears mentioning is that the present method works best when the number of entries in the trajectory is much larger than the number of dimensions. This condition helps ensure that the high-dimensional probability distribution can be estimated well. This limitation is overcome for a lot of chemical systems by virtue of underlying low-dimensional data structure or presence of correlated changes among the observables. While one might expect that in order to accurately estimate these probability distributions, one needs *n* ≫ 2^*d*^ data points in a *d*-dimensional data set, when the intrinsic dimensionality of the system *d*′ is lower, *n* ≫ 2^*d*^′^^ may be sufficient. In addition, the nature of MD simulations further mitigates this issue—larger (i.e., higher-dimensional) systems require more simulation time (i.e., more data) in order to achieve convergence. Additionally, metrics adaptive to nonlinear but intrinsically low-dimensional manifolds are a topic of ongoing work. Dimensionality scaling of the method is evaluated by clustering the valine dipeptide data set based on 37 heavy-atom interatomic distances and, as mentioned above, clustering the HP35 data set based on 66 backbone dihedral angles.

## 2 Methods

**Figure 2:**
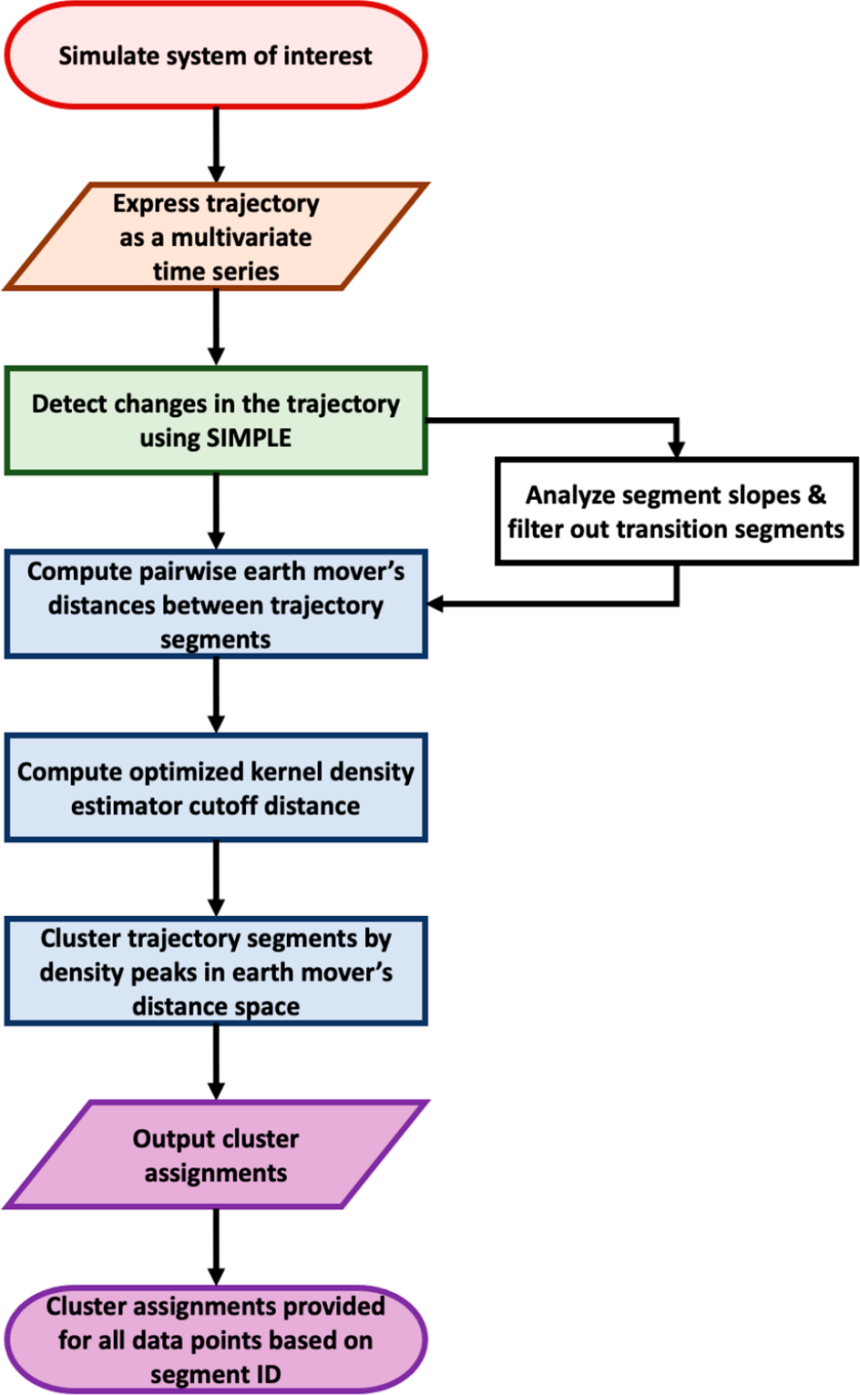
A schematic overview of the CATBOSS protocol.

A schematic overview of CATBOSS is presented in Figure 2.

### 2.1 Synthetic Data Set

**Figure 3:**
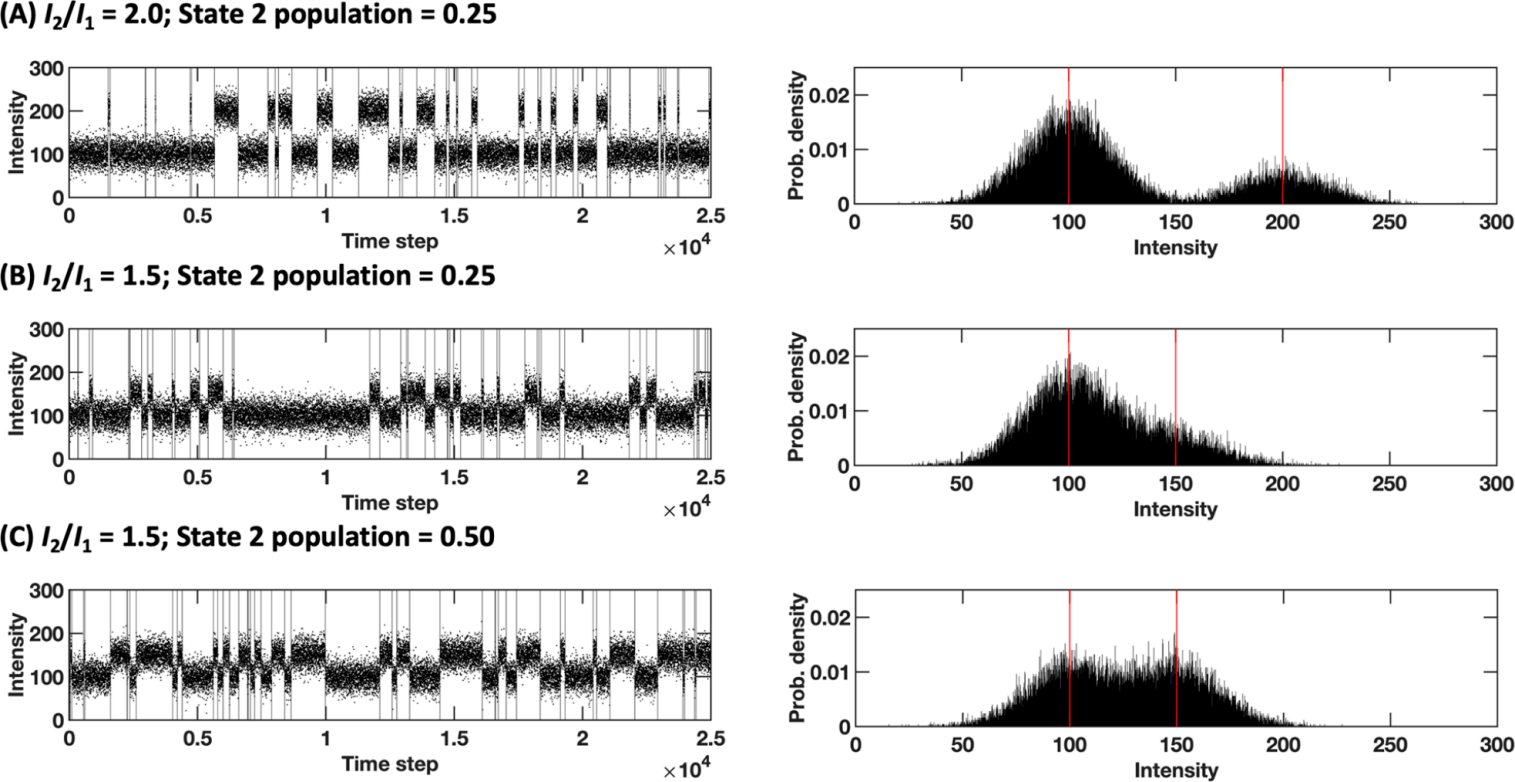
(A) A two-state, 1D model synthetic trajectory with the intensity ratio of 2.0 (“easy”) and state 2 population of 0.25 (“moderate”). (B) A trajectory with intensity ratio of 1.5 (“moderate”) and state 2 population of 0.25 (“moderate”). (C) A trajectory with intensity ratio of 1.5 (“moderate”) and state 2 population of 0.50 (“easy”). The left figure for each panel shows the time series view of the trajectory with ground-truth changes indicated. The right figure for each panel shows the data distribution with the mean values of each distribution marked by red lines. Note that the distribution in panel (B) approaches a unimodal appearance; separating the data set into the two underlying distributions may be difficult or imprecise to achieve by point-based clustering, as in Figure 1.

In a fashion similar to the testing methodology provided in Li and Yang’s work,^38^ we randomly generated 10 replicate data set batches. Each data set is a 25,000-step trajectory containing 50 segments sampled from 2 states (*N*_points_ = 25,000; *N*_segments_ = 50; *N*_states_ = 2). The state with the smaller population was referred to as the minor state (or state 2), and the population of state 2, that is, the fraction of the total population accounted for by the minor state was set to between 0.05 and 0.50 in increments of 0.05. The intensity (i.e., the mean of the distribution the data were drawn from) of the major state (*I*_1_) was fixed at 100; the intensity ratio between the minor and major states (*I*_2_/*I*_1_) was between 1.02 and 2.00, in increments of 0.01 between 1.02 and 1.05, and 0.05 thereafter. The standard deviations of both the major and minor states were fixed at 20 (σ_1_ = σ_2_ = 20 (see Figure 3 for example trajectories). Segment lengths were randomly generated so that their sum would equal the corresponding state’s population, with a minimum segment length of 5. We compared the performance of our method to that by Li and Yang,^38^ first by running the protocols in their entirety, that is, using their change-point detection algorithm and their clustering algorithm and then by running their clustering algorithm only, while providing the ground-truth change points.

### 2.2 Simulation Systems and Protocol

**Figure 4:**
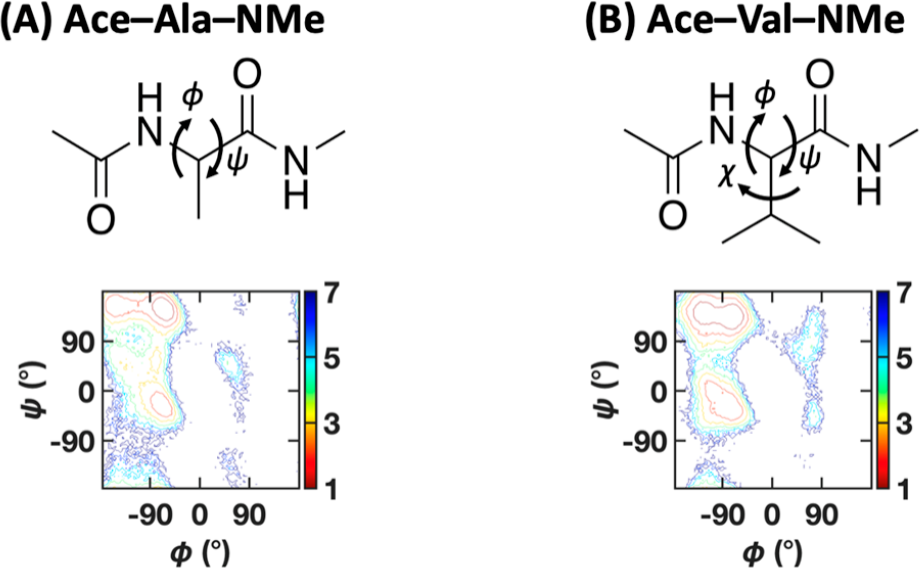
Model peptide systems. The structure (top) and the Ramachandran plot from the MD simulation (bottom) of (A) alanine dipeptide and (B) valine dipeptide. The contours are free energy levels separated by 1 *k*_B_*T*.

The two model dipeptides simulated by our group, Ace-Ala-NMe (alanine dipeptide) and Ace-Val-NMe (valine dipeptide) (Figure 4), were simulated using conventional MD performed in the GROMACS software suite.^44^ The RSFF2 force field, parametrized using a coil library with the goal of recapitulating intrinsic (ϕ, ψ) preferences of amino acids, was used with the TIP3P water model.^45,46^ Simulation convergence was verified by performing two sets of simulations for each system, starting from distinct initial structures. Each initial structure was energy-minimized, solvated, and equilibrated, after which a 200 ns simulation of alanine dipeptide and a 250 ns simulation of valine dipeptide were performed. The *NPT* production runs were conducted at 300 K and 1 bar, with a 2 fs time step. Peptide coordinates were saved every time step. Upon conclusion of the simulation, convergence was verified by calculating the normalized integrated product^47^ of the two simulations’ (ϕ, ψ) density profiles, which was found to be equal to 0.99 in both cases. One of the two simulations was then used for subsequent analysis. More details of the simulation setup can be found in the Supporting Information. The resulting Ramachandran plots were also compared to those reported in the original RSFF2 paper and found to be in close agreement (Figure 4).^45^

From the raw atomic coordinates, the backbone dihedrals ϕ and ψ were calculated for alanine dipeptide. Past dimensionality reduction studies have shown that these two dihedral angles map well to the intrinsic coordinates of the system, as the longest relaxation time components of the system’s motion.^48−50^ For valine dipeptide, three data sets were constructed: (1) one containing the backbone dihedral angles ϕ and ψ, (2) one containing those two angles, as well as the side chain dihedral χ, and (3) one containing the heavy-atom interatomic distances, with atom pairs only one or two bonds apart removed as well as pairs of atoms known to be coplanar due to their positions across the peptide bonds removed. The last data set had 37 dimensions. It is known that valine dipeptide exhibits three distinct side chain rotamer states: χ = 60°, χ = 180°, and χ = 300°.^51^ These three side chain conformations each have distinct backbone geometry preferences, and typically, the angle χ must be considered as part of structural analysis.^51^ Interatomic distances were used to confirm the higher-dimension scaling of the CATBOSS method, allowing for analysis without *a priori* knowledge of underlying low-dimensional structure, as well as to verify that no other important peptide degrees of freedom were neglected.

**Figure 5:**
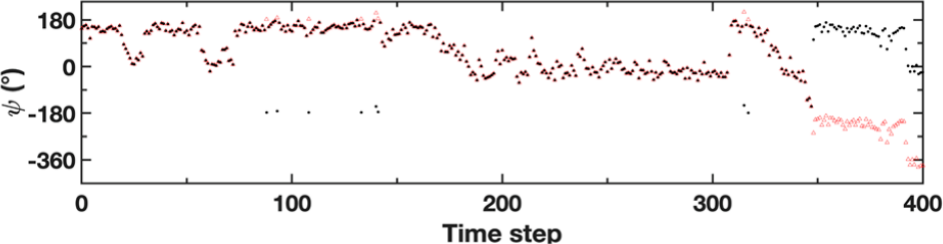
An example of trajectory shifting to preserve distance upon crossing the periodic boundary. The raw trajectory is shown in black dots. The trajectory is shifted (red triangles) when two consecutive points are more than 180° apart.

The 2 fs dihedral angle trajectories were shifted whenever the periodic boundary was crossed (e.g., a change from 179° to −179° would become a change from 179° to 181°, to reflect the true magnitude of the change), to avoid falsely detected change points. The shifting process is illustrated in Figure 5. The shifted trajectories were subsequently subsampled to a 1 ps sampling rate, for a total of 200,000 and 250,000 data points for alanine dipeptide and valine dipeptide, respectively.

In addition to these two systems, we also considered two long-time scale MD trajectories of protein systems. The BPTI trajectory was generously provided by DE Shaw Research.^12^ This trajectory contains approximately 1.03 ms of total simulation time, with a 25 ns sampling rate, accounting for 41,250 frames, and served as one of the data sets used to validate Sapphire-based clustering, another recently published method that aims to preserve kinetic data by taking advantage of the temporal character of noisy time series.^41^Sapphire-based clustering uses the progress index algorithm^52^ to group similar frames together and computes a kinetic annotation variable based on the transition counts for each progress index.^41^ To ensure a fair comparison, we apply the same manual featurization process as Cocina et al.^41^ PyEMMA 2.5^53^ was used to select all the backbone and side chain dihedrals. The side chain dihedrals for each of the three disulfide bridges in BPTI were added, and dihedrals corresponding to symmetric or fixed substituents (χ_2_ on Phe, Tyr, and Asp residues and χ_3_ on Glu and Tyr residues) were then manually removed, yielding 271 dihedrals remaining.^41^ The sines and cosines of these dihedrals were then processed using time-structure-based independent component analysis (tICA)^54,55^ with a lag time of 500 ns. The top 10 components were used for further analysis. We performed structural analysis of our cluster results and used PyEMMA to build a Markov state model (MSM) with a lag time of 500 ns, based on CATBOSS state assignments. This MSM was then validated using a 10-fold cross-validated VAMP2 score calculation.^56^ Under the VAMP framework, a Markov process is described by the Koopman equation.^57^ The top singular values of the Koopman operator (corresponding to the slowest modes of the process) can be optimized and summarized in a score value, which can be used to compare different trajectory discretizations.

The HP35 trajectory was also provided by DE Shaw Research.^42^ The trajectory contains approximately 300 μs of simulation time, with a sampling rate of 200 ps and 1,526,041 frames. This data set has also served as a benchmark data set for a clustering protocol accounting for kinetic information—the most probable path (MPP) clustering algorithm published by Jain and Stock.^43^ This algorithm relies on an initial structural discretization of the trajectory space using *k*-means, followed by a kinetic-oriented stage, which assumes a time scale separation between intra- and interstate transitions. At this stage, structural microstates with a self-transition probability below a chosen threshold are merged with their most probable transition, until a state whose highest transition probability is that of self-transition is reached.^43^ Again, in order to compare CATBOSS to this algorithm on even grounds, we followed the same featurization protocol: We began with backbone dihedral angles ϕ and ψ of residues 2–34 (excluding the terminal residues) and applied dihedral principal component analysis (dPCA)^58^ to reduce dimensionality. Eleven multipeak dPCs were used for analysis. We present an evaluation of cluster structures as well as the corresponding MSM. To demonstrate high-dimensional scaling of CATBOSS, we also applied our protocol to the same data set, without any dimensionality reduction of the 66 backbone dihedrals.

### 2.3 Change-Point Detection

**Figure 6:**
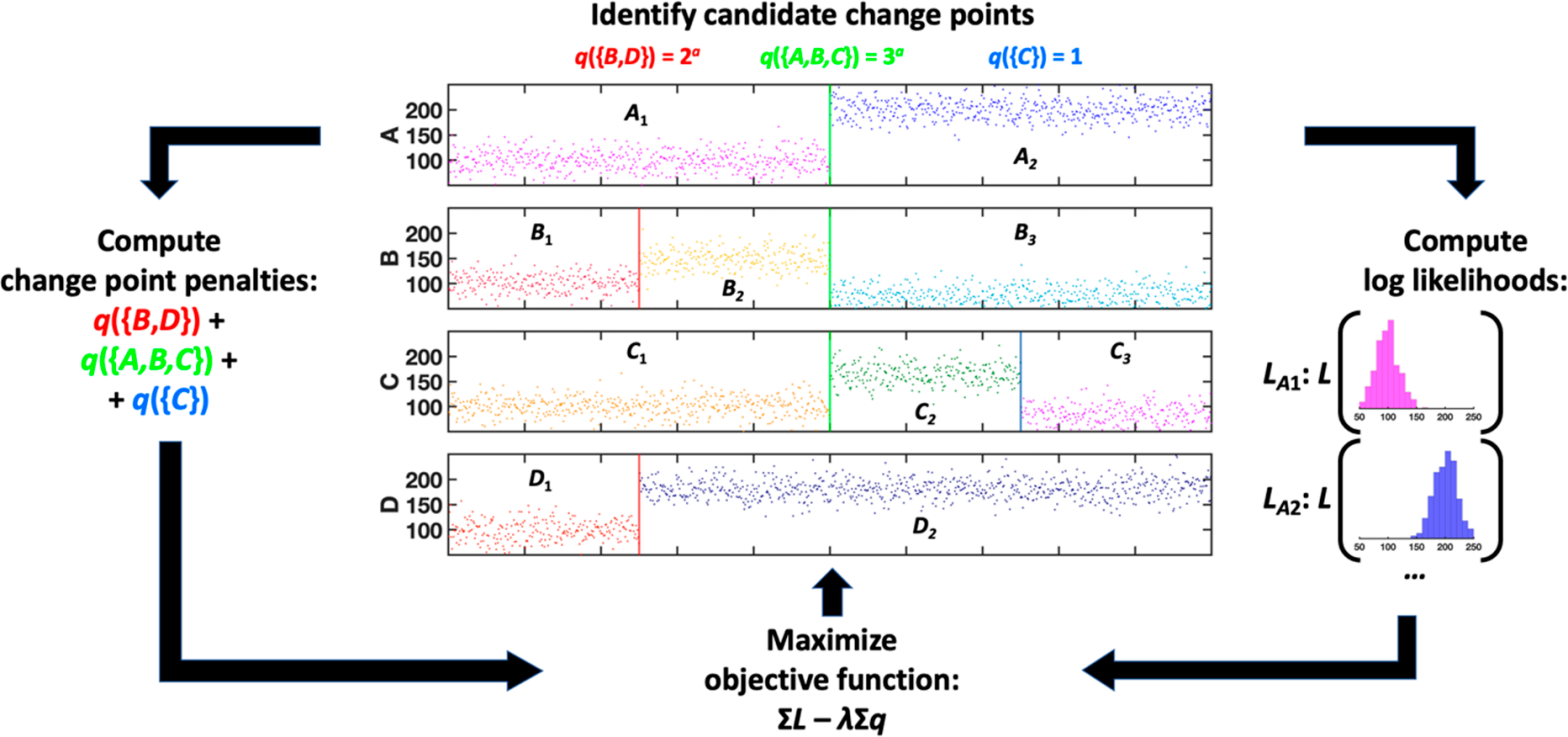
Schematic overview of the SIMPLE change detection algorithm.^39^ The local penalty at each time point depends on the number of changes detected and the simultaneity parameter α.

Change detection was performed using the SIMPLE change detection algorithm implemented in Python.^39^ The main principle of the algorithm is illustrated in Figure 6. SIMPLE relies on the underlying idea that within a noisy time series typical of an MD trajectory, points between two successive changes will be sampled from the same distribution. It is further motivated by the assumption that neighboring regions of a molecule are likely to undergo conformational changes simultaneously.^39^ SIMPLE outputs a set of change points *S*, which maximizes an objective function of the form:
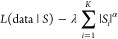
1where *L*(data | *S*) is the log-likelihood of data given the selected set of change points, and λ∑_*i* = 1_^*K*^ |*S*_*i*_|^α^ is a penalty function. In the penalty function term, |*S*_*i*_| is the number of changes detected in all variables at time *i*, λ > 0, and 0 ≤ α ≤ 1. For any candidate set of change points, the data are fit to a family of distributions defined by mean and spread; fitting to a family of distributions ensures that the method is translation and scaling invariant. The log-likelihood values are added over all data segments, for all observables. The implementation of the algorithm used here fits the data to a Laplace distribution family, where the log-likelihood is given by
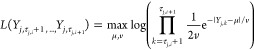
2where μ is the mean of the distribution, and *v* is the scale parameter. For this distribution model, the SIMPLE optimization problem has been shown to be asymptotically consistent.^39^

The penalty function, the intensity of which is tuned by the user-set parameter λ, prevents overfitting by balancing out the first term, which increases as additional change points are added. The other tuning parameter, α, affects the extent to which the penalty is lessened for changes occurring simultaneously across multiple variables, with 1 being no change to the penalty, and 0 being no additional penalty past the first changing variable.^39^ The default parameter values, λ = 20 and α = 0.7, were applied to the interatomic distances data set for valine dipeptide, and the parameter values of λ = 100 and α = 0.7 were applied to the long HP35 trajectory. For the remaining data sets, a range of λ values between 10 and 20 was tested, yielding similar results; λ = 10 was chosen out of an abundance of caution—generally speaking, subsequent clustering can “rescue” false positive change points (though excessive splitting may diminish the amount of kinetic information preserved), but not false negative ones. Similarly, α values of 0.7 and 1.0 were both tested, with differences between the two found to be minor, particularly in the low-dimensional data sets. The α value of 0.7 was chosen in line with the original paper’s guidance and in keeping with chemical intuition that conformational changes are often driven by coupled motions in multiple degrees of freedom.^59^

In a more general scenario, depending on the system and the trajectory sampling rate, additional parameter tuning may be necessary. An appropriate first step involves applying SIMPLE systematically, starting with a very high initial value for λ (i.e., capturing the most “obvious” changes) and decreasing the value on a logarithmic scale until the desired (or if unknown, a reasonable) change time scale is observed.^39^

### 2.4 Calculating Intersegment Distances

**Figure 7:**
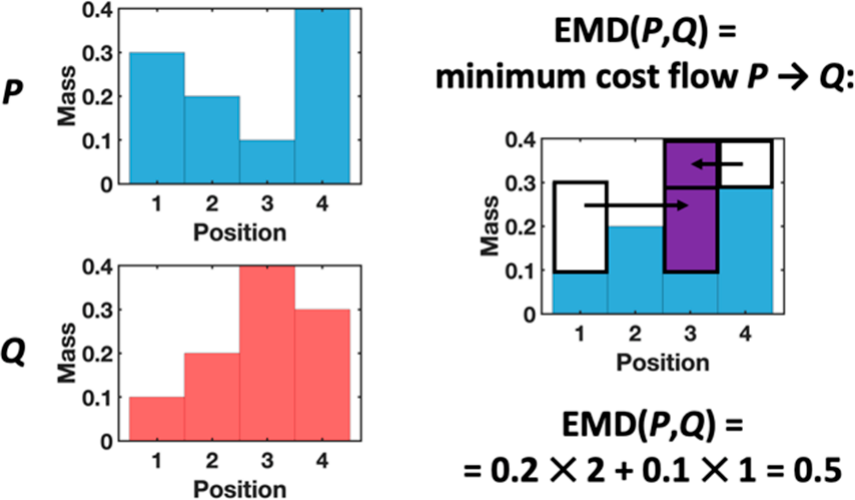
Schematic overview of the earth mover’s distance metric (partially motivated by reference (84)). Cost or “work” is defined as the mass times the distance moved; bars are given unit width so mass can be read directly off the *y* axis.

After trajectory segments were determined by SIMPLE, the distances between pairs of segments were calculated using the earth mover’s distance metric implemented in MATLAB.^40,60^ This distance metric, illustrated in Figure 7, presents a transportation problem solved by determining the minimum cost flow between two histograms, that is, the minimum work needed to transform one histogram into the other (in other words, to turn one “pile of dirt” into another, hence the name). In this case, work is defined as the amount of “dirt” moved times the distance by which it is moved. Earth mover’s distance has previously been applied as a metric of conformational similarity between free energy landscapes,^61^ but has not, to our knowledge, been used for clustering MD data. Earth mover’s distance between two histograms *P* and *Q* is given by
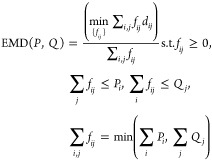
3where {*f*_*ij*_} are the flows and *d*_*ij*_ are the ground (in our case, Euclidean) distances between bins *i* and *j* of histograms *P* and *Q*, respectively. The probability masses were set to be equal, with segments length-normalized such that ∑_*i*_*P*_*i*_ = ∑_*j*_*Q*_*j*_ = 1, and the distances computed using Pele and Werman’s FastEMD package.^60^ The pairwise distances between the segments were organized into a distance matrix and used as the input for density-peak clustering, with local density contribution of each segment set to its length, given in number of data points.

The choice of earth mover’s distance was motivated by several reasons: Compared to less computationally intensive metrics, such as Jensen–Shannon divergence^62^ or normalized mutual information,^63^ earth mover’s distance is more general, as it does not require the probability distributions being compared to have overlapping domains. For a simple example, consider the idea of distributions supported on parallel line segments in : suppose *P*_*θ*_ is the distribution of , where *Z* is a random variable and θ is a fixed parameter. Then the earth mover’s distance EMD(*P*_0_, *P*_*θ*_) = |θ|, and is therefore continuous and converges to zero as θ → 0. Commonly used divergences such as Jensen–Shannon and Kullback–Leibler,^64^ are discontinuous at 0: JS(*P*_0_, *P*_θ_) = log(2), θ ≠ 0, and similarly KL(*P*_0_, *P*_θ_) = ∞, θ ≠ 0.^65^ Thus, earth mover’s distance captures the intuition that *P*_θ_ is getting “closer” to *P*_0_ as |θ| decreases, unlike Jensen–Shannon and Kullback–Leibler. More precisely, the topology induced by the Jensen–Shannon and Kullback–Leibler divergences is extremely strong, which is particularly problematic when the distributions being compared do not have overlapping supports.^65^ As a “weaker” metric, earth mover’s distance is also better suited to learning distributions supported by low-dimensional manifolds, which is often relevant for systems studied by MD simulation.^65^ Additionally, earth mover’s distance is more intuitive, as it has the units of the observables being compared. Its computational drawbacks can also be mitigated through quasilinear-time approximations.^66^ Further, compared to Fréchet distance, another *p*-Wasserstein (*p* = 2) distance (note that, strictly speaking, the equivalence between Fréchet distance as defined on curves and 2-Wasserstein distance holds true only when the curves are densities) previously used to evaluate trajectory similarity,^67^ earth mover’s distance (*p* = 1) is more robust to the presence of outliers in the probability distributions being compared. The modular character of CATBOSS does, however, allow for an easy application of other distance metrics.

### 2.5 Clustering by Density Peaks

The trajectory segments were clustered using a modified implementation of density-peak-based clustering by Rodriguez and Laio.^27^ Under the density-peak clustering scheme, each segment is assigned a local density ρ, computed using a Gaussian kernel, and the distance δ from the nearest neighbor of higher density. Points with ρ and/or δ substantially greater than the majority of points were selected by inspection as putative centroids. Cluster assignment was performed in a single set of operations, with each segment assigned to the same cluster as its nearest neighbor of higher density.^27^ The principal algorithm was left largely intact, with modifications limited to preallocating memory for execution speed, and allowing the selection of a nonrectangular region on the decision graph. As previously mentioned, density-peak-based clustering relies on a hardcoded distance cutoff for the kernel density estimator. In the original implementation, this value is set to a fixed (second) percentile of the sorted list of pairwise distances. While the algorithm is fairly robust to cutoff choice, a list-position based cutoff may present issues with clusters of varying densities.^35^ In order to include information from all data points, while minimizing user input, for all segment-based clustering trials, the kernel density estimator cutoff was set to the average distance to the ln(*N*)-th nearest neighbor, where *N* is the number of trajectory segments considered. This choice of cutoff was motivated by the idea that the number of nearest neighbors *k*(*N*) must adapt to the underlying data distribution as the number of samples *N* → ∞.^68^ Indeed, one must consider *k*(*N*) → ∞ as *N* → ∞ to prevent degeneracy, but one must also have *k*(*N*)/*N* → 0 as *N* → ∞ to ensure locality, otherwise small-population clusters may be drowned out.^69^ We see that *k*(*N*) = ln(*N*) satisfies this property, albeit other choices may also give good results for our approach (e.g., *k*(*N*) ).

Point-based density peak clustering was also performed as a control on the dipeptide data sets. As saving all pairwise distances for data sets of this size (necessitating distance matrices of approximately 240 GB for alanine dipeptide, and 375 GB for valine dipeptide) was intractable, point-to-point distances were not saved and were instead generated on the fly, during the clustering. The distance cutoff was set, per the authors’ original code, to the second percentile of the sorted list of distances; however, instead of precomputing the full list of distances, a list of distances was generated for trajectories subsampled to 50 ps and used to determine the distance cutoff. In order to isolate the cutoff effect, point-based clustering was also performed using the same cutoff optimization scheme used for CATBOSS.

The density-peak algorithm defines cluster border regions as points within the density cutoff distance from another cluster. Points with a density below that of the highest-density border point are considered halo points and may be treated as noise.^27^ Results were obtained with and without halo control (i.e., removal of halo points from classification), in order to evaluate the method’s level of confidence and outcome of including potential noise in the results.

### 2.6 Identifying Transition Segments by Analyzing Slopes

**Figure 8:**
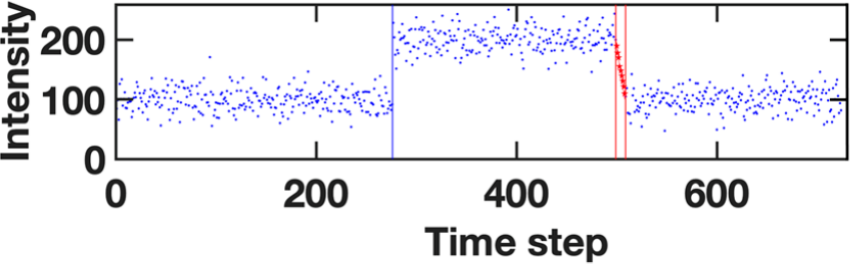
An example trajectory snippet showing the transition phenomenon. The blue vertical line shows a clean, abrupt transition typical of a change occurring on a time scale faster than the sampling rate. The red segment corresponds to a transition on a time scale slower than the sampling rate—a segment with a nonzero slope (shown by the red trend line) is detected.

As the sampling rate of the data set may occasionally be finer than the time scale on which conformational changes occur, we may see a MD trajectory data set contain points which do not actually belong to a metastable state but constitute an ongoing transition (see an example in Figure 8). Under the scope of CATBOSS, the set of trajectory segments found by SIMPLE includes segments consisting of such points. If we represent the MD trajectory as a time series, a metastable state will be a “flat” segment, consisting of points drawn from a distribution centered around a mean value (Figure 8, blue dots). On the other hand, a transition will be a segment with a distinct slope, connecting from one metastable state to another (Figure 8, red stars). We formulate a simple statistical test for a “sloped” segment. We begin with the idealized assumption that a trajectory segment of length *n* is drawn from a normal distribution with mean μ equal to the mean of the segment. Under this model, the slopes of best-fit regression lines for segments of length *n* drawn from this distribution will also be normally distributed, with the distribution mean equal to zero. We compute an empirical approximation of the standard deviation from the variance of residuals and the variance of the segment to be analyzed:
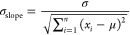
4where *x*_*i*_ is the *i*-th point of the segment, and σ is the standard deviation of the residuals in the regression model.^70^ For each segment, we calculated the slope of its best-fit regression line and compared it to σ_slope_ for that segment; if the absolute value of the slope was >1.96·σ_slope_ (*p* < 0.05), then we rejected the null hypothesis that the segment may be flat. This analysis was performed on the alanine dipeptide data set to illustrate our protocol’s ability to identify transition segments—something that currently available clustering algorithms struggle with. More specifically, while the density-peak algorithm includes a way to identify regions where the uncertainty in the density estimate is high (in the form of halo control),^27^ we observe that the use of this feature frequently results in over- or underestimation of transition regions. Figure S1 shows one such case, where unclassified points (shown in black) account for the majority of the data set. Similar results hold true with an updated density-peak protocol which merges clusters consisting entirely of points whose density is comparable to the border density with their neighboring clusters.^37^

The code for the protocol described above is available at https://github.com/ysl-lab/CATBOSS.

## 3 Results and Discussion

### 3.1 CATBOSS Reliably Separates Two Overlapping States of Varying Populations

**Figure 9:**
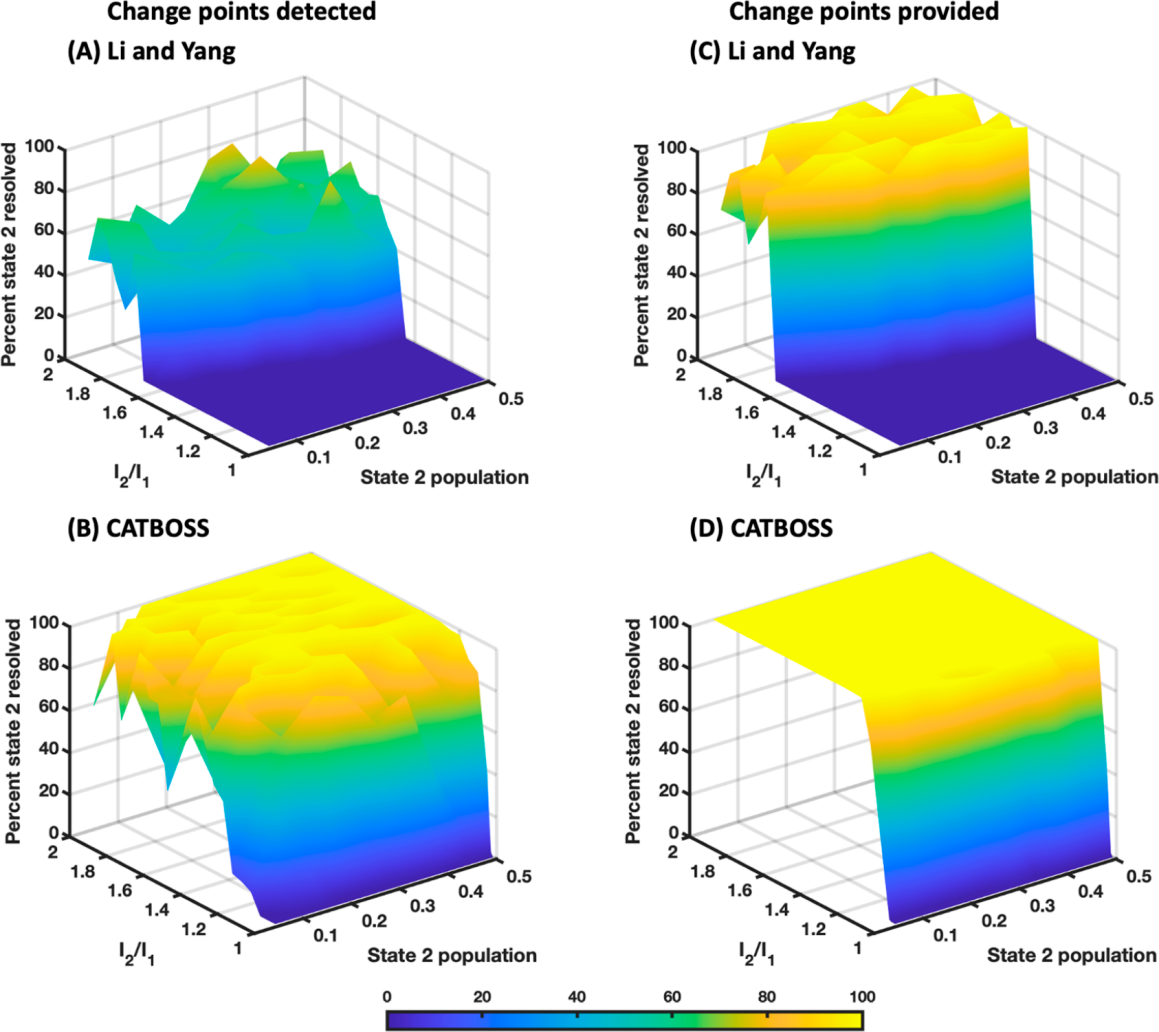
Accuracy of segment-based clustering methods on two-state, 1D synthetic data. (A) Li and Yang’s hierarchical method.^38^ (B) CATBOSS. (C) Li and Yang’s hierarchical method with ground-truth change points provided.^37^ (D) CATBOSS with ground-truth change points provided.

A summary of clustering results and comparison of CATBOSS with a recent segment-based clustering algorithm^37^ is given in Figure 9. The surface plot of clustering accuracy with respect to the intensity ratio (*I*_2_/*I*_1_) and state 2 population recapitulates the “curved waterfall” shape seen in Li and Yang’s work.^38^ Clustering accuracy in this case was defined as the percentage of total points belonging to the minor state that were correctly classified. As *I*_2_/*I*_1_ decreases, the two states display higher overlap and eventually they can no longer be distinguished (this phenomenon would occur even sooner in the point-based regime).^38^ On the other hand, as the population of the minor state decreases, the minor state population becomes significantly smaller than that of the major state, and the lowly populated minor state is ultimately overpowered by the highly populated major state and cannot be resolved, either. We note that CATBOSS consistently outperforms the other method in terms of peak accuracy, robustness to overlap, and robustness to population difference (Figure 9).

To test whether the performance difference between the previous method and CATBOSS was entirely attributable to change detection differences or not, we applied both algorithms to segments delimited using the ground truth change points (Figure 9C,D). It is found that CATBOSS outperforms the other protocol under these circumstances as well.

We notice that the difference in accuracy between the protocol adopted by Li and Yang and CATBOSS stems not only from the difference in the way that clustering is performed (hierarchically versus by density peaks) but also the final step in Li and Yang’s protocol, which uses the Bayesian information criterion to determine the most likely number of states.^38^ This statistical metric yields highly conservative results, leading to the determination of one lone cluster even when a higher number of clusters would yield correct assignments. In order to account for this, we also performed a round of tests in which the dendrogram was manually cut at two clusters; the results are shown in Figure S2. The performance in this case is very similar to that of CATBOSS; however, in a real-world use case, the number of clusters is not known in advance. In comparison, CATBOSS selected centroids based on a gap in ρ·δ, with decision graph assignments displayed and verified manually.

### 3.2 Compared to Point-Based Clustering, Segment-Based Clustering of Alanine Dipeptide Yields Natural State Boundaries

**Figure 10:**
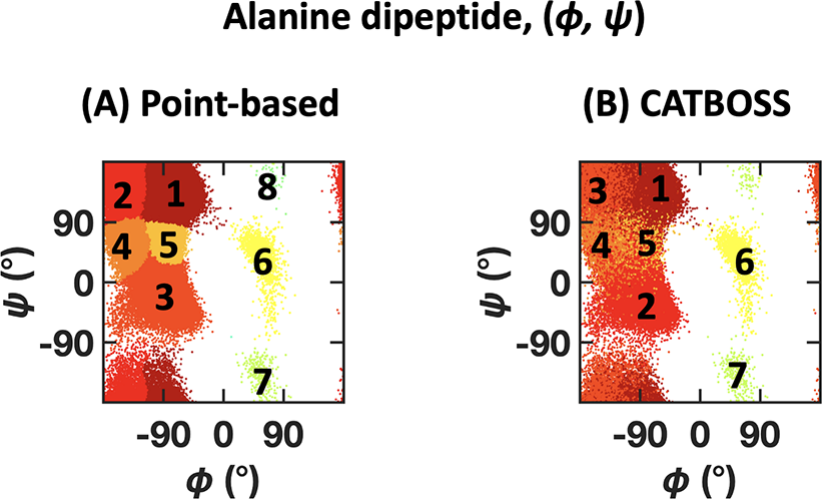
Performance comparison of (A) point-based clustering and (B) CATBOSS on the alanine dipeptide (ϕ, ψ) data set. The six most populated clusters for each method are shown as density contour plots in the Supporting Information.

**Figure 11:**
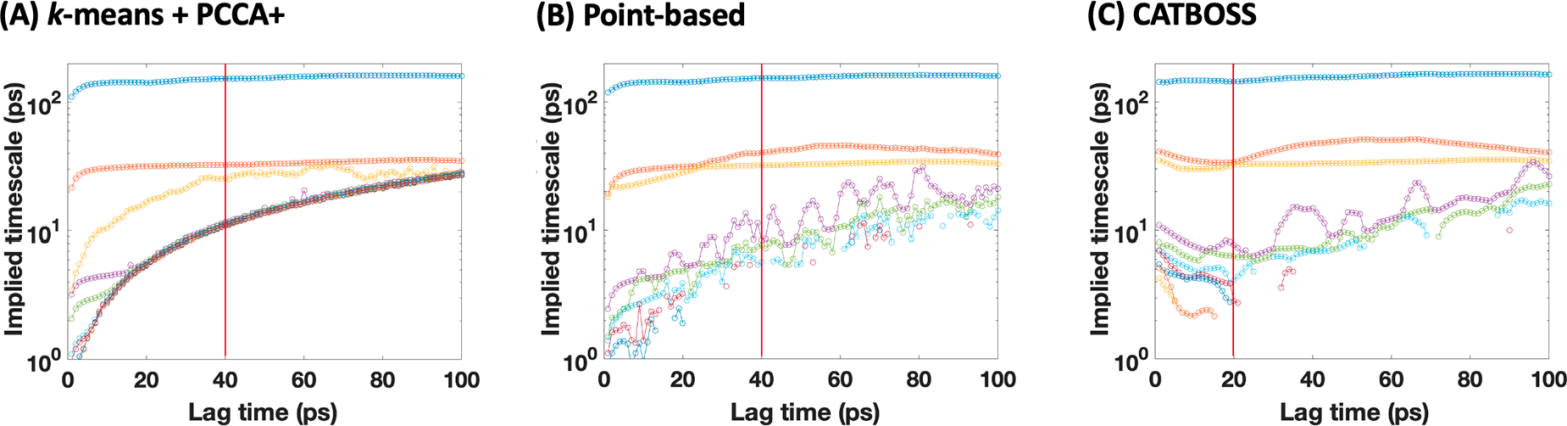
Implied time scale comparison on the alanine dipeptide data set for three different trajectory discretizations: (A) *k*-means structural clustering followed by PCCA+ kinetic clustering, (B) point-based density-peak clustering, and (C) CATBOSS. Note that the plateau occurs sooner in the case of CATBOSS.

A summary of clustering results on the (ϕ, ψ) trajectories of alanine dipeptide and comparison of CATBOSS with the point-based control is given in Figure 10. Alanine dipeptide is a simple, well-studied system, frequently used as a benchmark for MD data analysis tools.^48,50,71−73^ Its dynamics have been shown to have a low-dimensional intrinsic structure and are well-explained using the two backbone dihedral angles (ϕ, ψ).^48−50^ Unsurprisingly, both point- and segment-based clustering are able to learn the intuitive partitioning of the dihedral data, with similar populations across the board (Figures 10 and S3). However, the nature of point-based clustering prevents it from accounting for overlap between states and accounts for less natural-looking state boundaries in the control (for example, comparing the boundaries between states in Figure 10). The definition of state boundaries is an issue which has been previously identified and tackled in a variety of ways;^74−76^ however, generally speaking, commonly used notions of “core sets of states” do not take advantage of temporal information during the clustering stage. In the case of CATBOSS, the boundaries are a direct consequence of the time series features and require no further postprocessing. To demonstrate that these boundaries are well-defined, we built a MSM for this trajectory using the cluster assignment from point-based density-peak clustering and from CATBOSS as the state input. As MSMs are commonly constructed using a combination of fine-grained *k*-means and various types of kinetic clustering,^77−79^ as a reference we also built a MSM from a *k*-means discretization with 100 microstates merged using PCCA+, a popular fuzzy spectral clustering method based on a Perron eigenvalue cluster, which generally results in a non-negative, nearly block-diagonal transition matrix.^79^Figure 11 shows that the slow implied time scales appear to plateau at much shorter lag times when the CATBOSS clustering results were used as the state input, suggesting that CATBOSS does capture some kinetic information about the system.

### 3.3 Slope Analysis Suggests That States 4 and 5 Are Transitional

**Figure 12:**
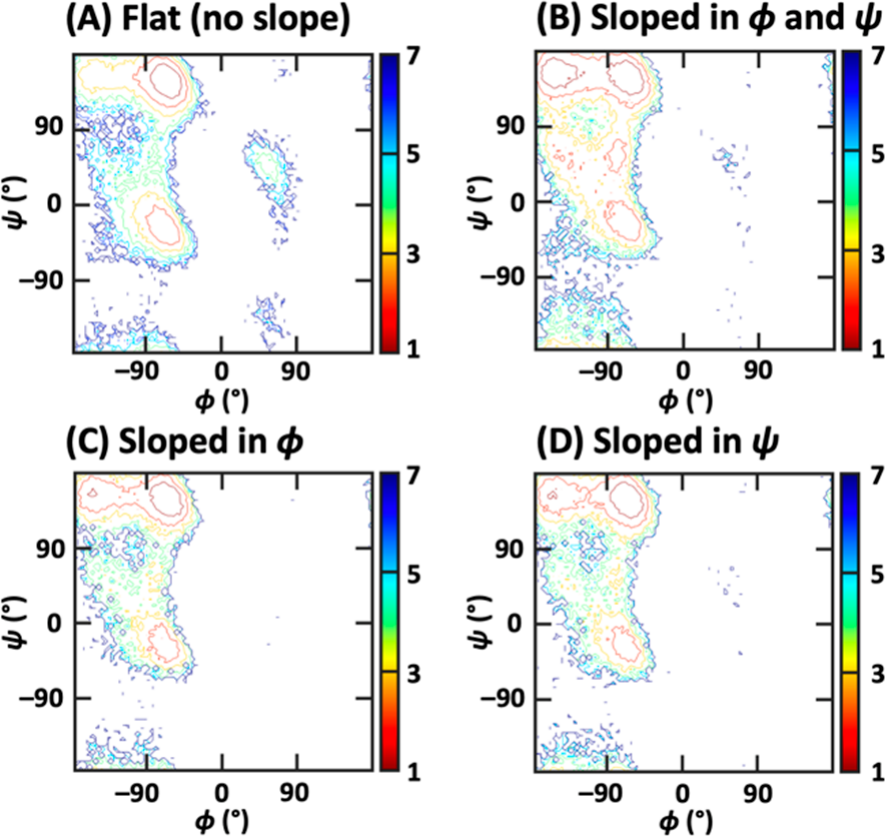
Free energy contour plots of data points from the alanine dipeptide data set belonging to segments classified by slope analysis. Note the overrepresentation of states 4 and 5 (Figure 10) among the sloped segments.

The state assignments in both the point-based and CATBOSS cases suggest the presence of non-negligibly populated states (states 4 and 5 in Figure 10; ∼3–4% in population) between the PPII/β region (states 1 and 2 in Figure 10A; states 1 and 3 in Figure 10B) and the right-handed α-helical region (state 3 in Figure 10A; state 2 in Figure 10B). Whereas point-based clustering provides little information about these states, clustering using CATBOSS shows that states 4 and 5 consist predominantly of short segments (Figure S4), suggesting short lifetimes despite the non-negligible state populations (∼3–4%). To further examine whether these states can be called “states”, implying that they are metastable, we applied the statistical test outlined in the Methods section to the alanine dipeptide trajectory. As Figure 12 shows, states 4 and 5 in Figure 10B were visibly overrepresented in the set of “sloped” segments. A similar observation was made for a number of segments initially grouped with state 3, potentially corresponding to transitions between states 1 and 3 in Figure 10B. To further substantiate these findings, a CATBOSS analysis was performed with the sloped segments removed from consideration. As Figure S5 shows, states 4 and 5 are no longer resolved as individual clusters.

### 3.4 Segment-Based Clustering of (ϕ, ψ) in Valine Dipeptide Reveals Additional Degrees of Freedom

**Figure 13:**
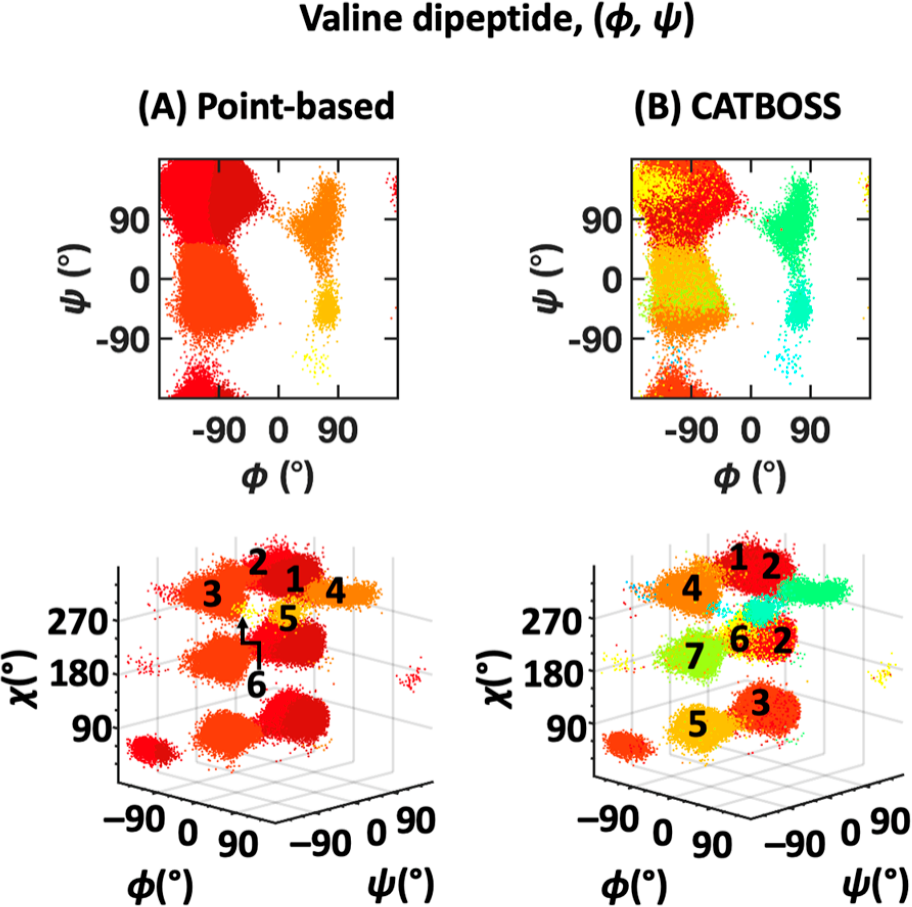
Performance comparison of (A) point-based clustering and (B) CATBOSS on the valine dipeptide (ϕ, ψ) data set. The six most populated clusters for each method are shown as density contour plots in the Supporting Information.

A summary of clustering results on the two-dimensional valine dipeptide data set, that is, using (ϕ, ψ), and comparison of CATBOSS with the point-based control is given in Figures 13 and S6. In the case of this system, the differences between CATBOSS and the point-based control are far more pronounced. CATBOSS yields a total of 11 clusters, compared to 6 seen in the point-based clustering. A look at the 3D plot of the data, with the side chain dihedral χ on the additional *z*-axis, reveals that to a large degree, the segment-based protocol was able to discriminate among different side chain rotamers, without being presented with the χ angle data (Figure 13, bottom row). This finding showcases a remarkable improvement in clustering resolution brought about by segmentation.

**Figure 14:**
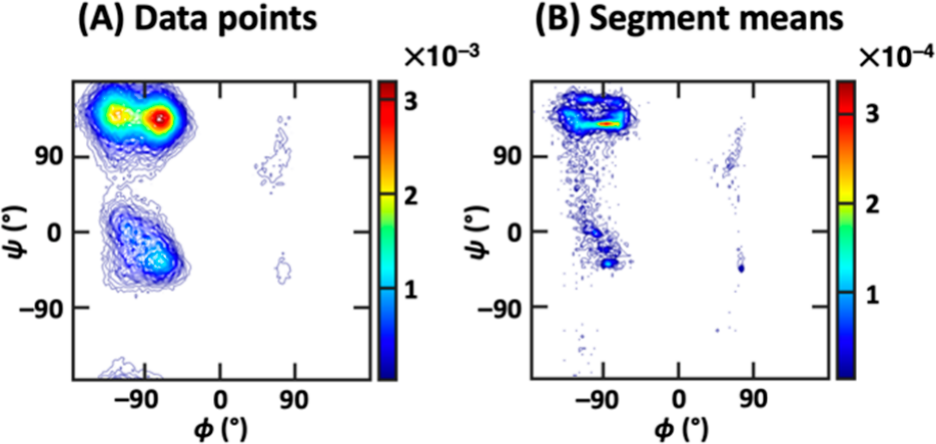
(A) All data points of the valine dipeptide data set projected onto the backbone dihedral space. (B) All segment means of the valine dipeptide data set projected onto the backbone dihedral space. Note the visible separation between subclusters in the major states in (B).

**Figure 15:**
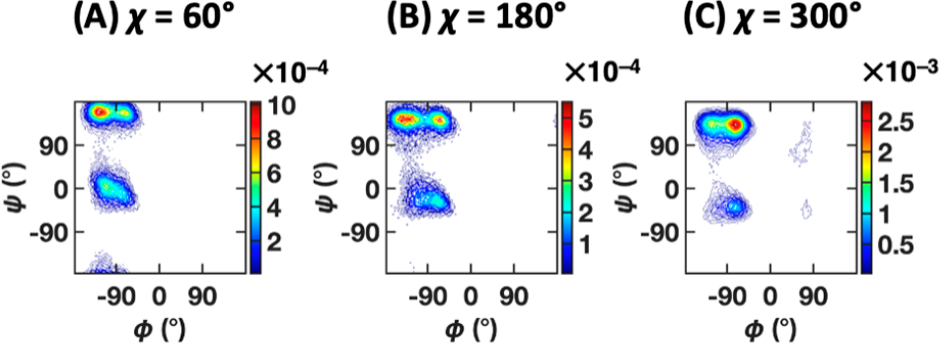
Density contour plots of subsets of the valine dipeptide trajectory corresponding to (A) χ = 60°, (B) χ = 180°, and (C) χ = 300°. Note the slightly different positions of the major state density peaks.

An illustration of the input of the two contrasting algorithms provides an explanation of the superior resolution of the segment-based algorithm: While the (ϕ, ψ) density distribution of the data points shows no subdistribution in each of the β, PPII, and α_R_ regions (Figure 14A), a look at the segment means readily shows further clustering, especially in the case of the β and α_R_ regions (Figure 14B). Such separation is impossible to see on the data-point level. This behavior may be a result of different side chain orientations inducing slight backbone conformational change, leading to a small-magnitude shift in the position of free energy minima. Figure 15 shows the backbone (ϕ, ψ) distribution for each of the three side chain conformers (χ = 60°, 180°, and 300°). The different positions of the density peaks in the β, PPII, and α_R_ regions are clearly observed and they are possible to capture using CATBOSS, due to the segmentation of the data emphasizing density gaps between states, while allowing for overlapping data distributions. It is worth noting, however, that there is not always a clear separation between the β and PPII regions (Figure 13B, bottom).

### 3.5 Including the Side Chain Dihedral Angle Results in Intuitive Partitioning

**Figure 16:**
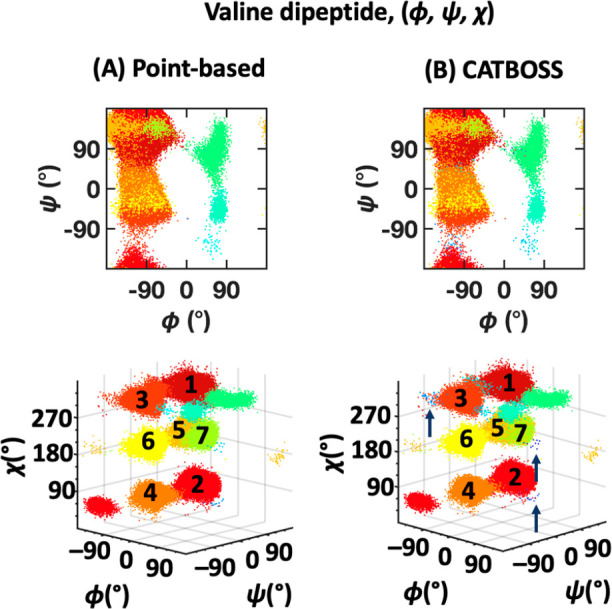
Performance comparison of (A) point-based clustering and (B) CATBOSS on the valine (ϕ, ψ, χ) data set. The six most populated clusters for each method are shown as density contour plots in the Supporting Information.

A result summary for the three-dimensional (3D) (ϕ, ψ, χ) data set of valine dipeptide is given in Figures 16 and S7. Including the side chain dihedral angle χ makes a clustering algorithm’s job “easier”, insofar as it provides most of the information about valine dipeptide’s behavior.^51^ With this information provided, as seen in the alanine dipeptide case, both point-based clustering and CATBOSS perform well, with relatively minor differences (Figure 16). Aside from boundary improvements, we also observe that CATBOSS singles out low-population regions (states marked in shades of dark blue in the 3D panel of Figure 16B, pointed at by dark blue arrows) on the periphery of major states that readily lend themselves to interpretation as transition segments analogous to those previously described for alanine dipeptide.

To further evaluate the obtained cluster assignments, we separated the data set based on the value of the side chain dihedral χ and applied both methods to each individual conformer separately. We then observed that when clustered individually, all conformers displayed good separation between the β and PPII regions (Figure S8). We believe this difference in separation to be the result of the structure in the valine dipeptide (ϕ, ψ, χ) data set. The data formed three well-separated “layers” in the χ space, corresponding to the three side chain conformers, wherein the larger average distances between points lead to a larger cutoff being selected. However, since points associated with the same side chain conformer have negligible distance in χ, the larger cutoff results in points separated by only small density valleys being lumped together when the entire data set is evaluated at once.

However, in the case of CATBOSS, when clustering each side chain conformer separately, in addition to the β and PPII regions being resolved separately, we obtain additional states with segment means between the two major regions and data points traversing both (Figure S8B, states 4–6 in the left panel, and state 4 in the middle panel). Visual inspection of the trajectories (Figure S9) suggests that the segments in question are bimodal, that is, sampling from more than one distribution. The presence of these segments hints at a change detection artifact. It is noteworthy that the transitions between the two modes occur at a very fast rate, comparable to our sampling frequency, which makes change detection more difficult. Observing both fast and slow interconversion between the two states may be due to interactions between the peptide and the solvent molecules.^80^

### 3.6 Interatomic Distance Clustering Provides Evidence of Scaling and Validation of 3D Data Set

**Figure 17:**
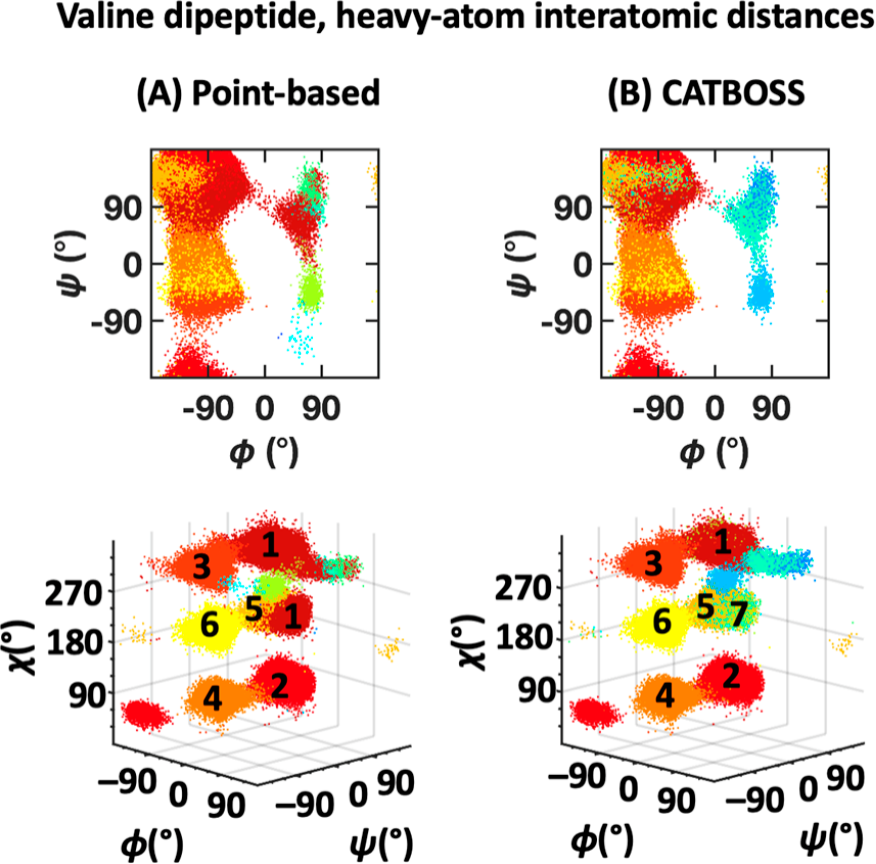
Performance comparison of (A) point-based clustering (all data shown, as noise removal discarded most data) and (B) CATBOSS on the valine dipeptide data set (85.5% data shown, following noise removal), considering heavy-atom interatomic distances. The six top population clusters are shown for each method, and the contour plots are shown as density contour plots in the Supporting Information.

The summary of results on the 37-dimensional data set of valine dipeptide is provided in Figures 17 and S10. As much as the inclusion of a third dimension made clustering easier, the consideration of all heavy-atom interatomic distances ought to make it more difficult, primarily by challenging the scaling of the distance metric used into higher dimensions.^81,82^ Good scaling is important for an algorithm to have widespread practical appeal, as oftentimes there is no *a priori* knowledge of intrinsic coordinates to consider, or there is no sufficiently low-dimensional intrinsic coordinate set that may explain a large enough portion of the data variance.^83^ In those cases, the use of a relatively (or entirely) complete set of internal coordinates may be warranted. While the Euclidean-distance point-based clustering scales reasonably well, some misclassifications (e.g., state 1) are still apparent, across multiple reasonable decision graph assignments (the perceived best one of which is shown in Figure 17A). We attempted manually optimizing the kernel density cutoff, to no avail, and enabling halo control with the applied cutoff, that is, removing noncore points from classification, resulted in the majority of data being unclassified. On the other hand, with the cutoff used, halo control had much more utility on the segment-based front, leaving 85.5% data classified, with no major deviations in assignment compared to the 3D data set (Figures 16B and 17B), leading to an overall more accurate clustering compared to the point-based control.

### 3.7 CATBOSS Provides a Good Description of the Folded Dynamics of BPTI

**Figure 18:**
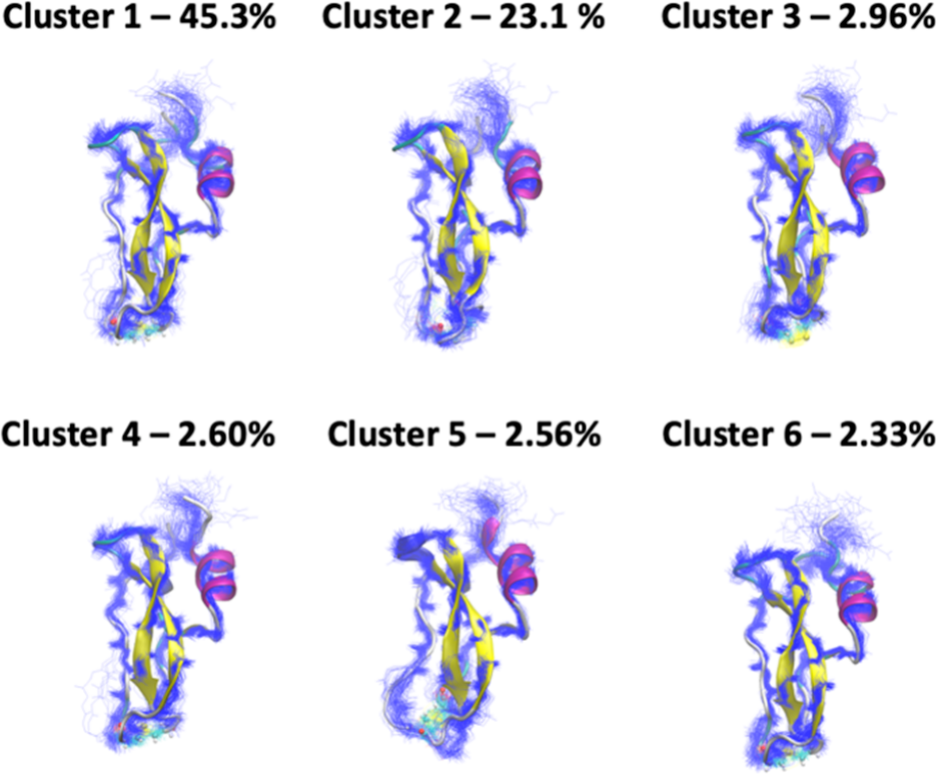
BPTI clustering results. 100 randomly selected representative structures of the top 6 clusters detected by CATBOSS for the BPTI trajectory. The Cys14-Cys38 disulfide bridge is shown as a ball and stick model.

A breakdown of the clustering results is presented in Figure 18, with the top 6 (out of total 34) clusters shown. The individual cluster distributions of tICs are sharp and unimodal (Figure S11), suggesting clean cluster separation. The tight backbone distributions (shown in blue lines in Figure 18) further corroborate this observation. As previously reported,^41^ the Cys14-Cys38 disulfide bridge (shown as a ball-and-stick model) does appear to be a prominent, though not the only discriminating factor between the clusters. The distributions of dihedral angles describing the geometry of this disulfide bridge are given in Figure S12. Considering the implied time scales of MSMs built at various lag times, we confirm that a lag time of 500 ns is appropriate for this state selection (Figure S13). With this lag time selection, a 10-fold cross-validated VAMP2 score was calculated with a validation fraction of 0.1 and the top 10 singular values accounted for.^56^ The resulting mean VAMP2 score was 4.45, which exceeds those attained by all the methods tested by Cocina et al. under the same settings.^41^ Additional parameter tuning may further improve upon this score.

### 3.8 CATBOSS Discriminates between Folded, Unfolded, and Intermediate States of HP35

**Figure 19:**
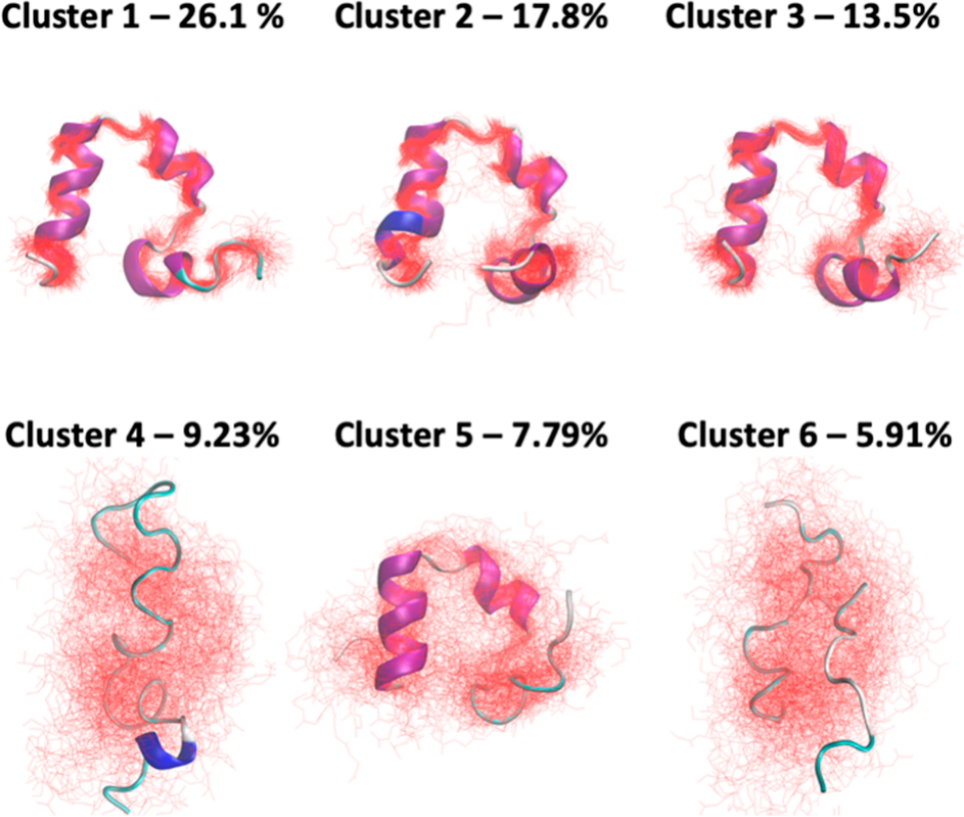
HP35 clustering results. 100 randomly selected representative structures of the top 6 clusters detected by CATBOSS for the HP35 trajectory. Note the presence of a folded state (cluster 1), intermediate states (clusters 2, 3, and 5), and unfolded states (clusters 4 and 6).

An overview of the CATBOSS clustering results is given in Figure 19, with the top 6 (out of 17) clusters shown. The per-cluster distributions of dPCs (Figure S14) are sharp and unimodal for the high-population clusters, supporting a clean partitioning. A visual examination of representative cluster structures (Figure 19) shows that the states observed range from native-like (cluster 1) to partially folded intermediates (clusters 2, 3, and 5), to entirely unfolded (clusters 4 and 6). This observation is in line with previous work.^43^ Based on the MPP results, Jain and Stock suggest that a subset of residues is particularly relevant to state discrimination; residues in positions 3, 9–13, and 29–33 appear to be where the primary differences between clusters are concentrated.^43^ More specifically, residue 3 assumes distinct conformations in native-like states, the unfolded state, and different intermediates, changes in residues 9–13 are associated with the unfolded–intermediate transitions, and residues 29–33 distinguish the two native-like states.^43^ Upon examination of the Ramachandran plots for the residues, it appears that that is indeed the case (Figures S15 and S16). We are similarly in agreement that there are multiple native-like and intermediate states with appreciable population, though some additional splitting of states is seen with CATBOSS assignments. The populations of native-like states (Figure S16) match up with the 31% figure reported by Jain and Stock following their dynamic coring procedure.^43^

To further demonstrate CATBOSS’ scaling capabilities, we repeated the above analysis for the 66-dimensional trajectory consisting of the raw backbone dihedral angles without any dimensionality reduction. Based on the dihedral distributions of each cluster (Figure S17), we see that the clustering remains robust, albeit with a different population distribution; it appears that native-like states are split further, with the top intermediate clusters now merged.

Overall, the results shown demonstrate the ability of CATBOSS to handle large data sets and process them to a more manageable form. With the SIMPLE parametrization used here, the 66-dimensional data set was reduced from 1.5 × 10^6^ frames to approximately 30,000 segments—a 50-fold decrease in data set volume with no apparent loss of information.

### 3.9 Remarks on Robustness

The presence of two tuning parameters at the change detection stage of the process, as well as the ability for the user to optimize the density cutoff distance and affect core/halo assignments may be perceived as the algorithm having a lot of “moving parts”. The plug-and-play potential of the method was confirmed by comparing the performance of the protocol with the parameter values shown here to that with a range of other values. The results using different values of SIMPLE tuning parameters are shown in Figure S18 and imply a high degree of robustness in this regard, with the caveat that in cases where finer separation is desired or extremely fast or slow transitions are present, more sensitive tuning may be advisable (Figure S19). Moreover, the newly introduced automatically set cutoff yielded not only strong performance across all data sets but also resulted in the majority of data being classified as core points, with a high degree of confidence. In fact, enabling halo control almost exclusively declassified points identified as belonging to transition segments, either by inspection or slope analysis. The default cutoff applied in the stock version of the point-based density-peak clustering code, on the other hand, yields robust assignments, but is not necessarily optimal for every system, and may result in highly uncertain halo control.

### 3.10 Remarks on Performance

One obstacle to the application of point-based clustering to truly large MD data sets was having to choose between quadratic memory complexity for storing pairwise distances and recalculating distances at every execution. With the stock MATLAB implementation, storing the distance matrix alone for a 200,000-point data set would require in excess of 370 GB of memory, even in single precision. With the settings described in the paper, the number of segments found by SIMPLE was on the order of 10^3^ for all data sets but alanine dipeptide and HP35, which clocked in at ∼10^4^ segments, corresponding to a worst-case distance matrix of ∼1 GB in single precision, which can be trivially stored for repeated executions as well as fit in the RAM of any modern computer. On the computational complexity side of things, no significant performance hit was observed as a result of SIMPLE preprocessing or earth mover’s distance calculation. In fact, given the need for repeated distance calculations in point-based mode, the segment-based method generally ran as fast or faster. Moreover, if further performance gains are desired and absolute accuracy may be sacrificed, we would like to point out that if the segments are not treated as histograms, but approximated by a continuous distribution with a given mean, variance, and probability mass, the earth mover’s distance is equivalent to the 1-Wasserstein distance, which may be analytically computed in quasilinear time for a single dimension.^66^ This approximation improves upon the “rate-limiting step” of the algorithm, allowing for efficient clustering of millions of entries.

## 4 Conclusions

Readily available computational resources and algorithmic improvements have turned MD simulation into an immensely powerful tool in a chemist’s arsenal. Cluster analysis is an essential component of the MD analysis workflow, vital to parsing an inevitably gigantic amount of data into a human-readable form. In this work, we have devised what we believe to be the first segment-based clustering protocol applied to MD data sets. This method improves on the performance of density-peak clustering and outdoes the state of the art by harnessing time evolution information to produce fuzzy state boundaries which are more consistent with systems’ dynamics and provide information on transitions. Most notably, we have presented evidence that segment-based partitioning greatly enhances the resolution of clustering and may uncover, or compensate for, the presence of hidden degrees of freedom. We have also presented a way to “pick out” transition segments and demonstrated robustness comparable to, or exceeding, the currently available techniques. The modular character of CATBOSS will allow it to further improve as adjustments are made to its components; for instance, incorporating slope analysis into the change detection stage may be a way to distinguish between metastable and transition states more robustly. Consequently, we expect that this method will be of great use to chemists seeking insight into molecules’ structural preferences and dynamics.

## 5 Data and Software Availability

The initial structures for all simulations performed, the simulation parameter files, all of the time series analyzed in this work, as well as all of the scripts necessary to perform the analysis, are provided free of charge at https://github.com/ysl-lab/CATBOSS. The README file for the repository provides links to third-party scripts used as part of the protocol. Full simulation trajectories of the dipeptide systems in .xtc format are available upon written request from the authors.

## Supporting Information Available

Additional details of simulation setup; performance of Li and Yang’s clustering algorithm on the two-state, 1D synthetic data with the number of clusters manually set to 2; results of alanine dipeptide clustering with halo control; Ramachandran plots of the top six clusters from the alanine dipeptide data set; CATBOSS cluster assignments when applied to flat segments of the alanine dipeptide data set only; Ramachandran plots of the top six clusters from the valine dipeptide data sets; cluster assignments of separately clustered χ conformers and within the 3D valine dipeptide data set; concatenated view of segments belonging to an artifact cluster in the 180° χ conformer subset of the 3D valine dipeptide data set; individual independent component distributions for the top three clusters of the BPTI data set detected by CATBOSS; distributions of the Cys14-Cys38 disulfide bridge dihedral angles for the top six clusters of the BPTI data set detected by CATBOSS; 10 slowest-relaxing implied time scales using CATBOSS-detected clusters of the BPTI trajectory as state input; individual dPC distributions for the top three clusters of the HP35 data set detected by CATBOSS; density contour plots of residues 3 and 9–13 for the top six clusters of the HP35 dPC trajectory clustered by CATBOSS; density contour plots of residues 3 and 29–33 for the top three native-like clusters of the HP35 dPC trajectory clustered by CATBOSS; full backbone dihedral distributions for the top six clusters (as determined by CATBOSS) of the raw HP35 backbone dihedral trajectory; CATBOSS cluster assignment dependence on the tuning parameter λ for the alanine dipeptide data set; CATBOSS cluster assignment dependence on the tuning parameter λ for the 3D valine dipeptide data set (PDF)

## Abbreviations

MD: molecular dynamics
CATBOSS: cluster analysis of trajectories based on segment splitting
BPTI: bovine pancreatic trypsin inhibitor
HP35: villin headpiece 35-residue subdomain
tICA: time-structure-based independent component analysis
MSM: Markov state model
MPP: most probable path
dPCA: dihedral principal component analysis
