# Supplementary material for: CATBOSS: Cluster Analysis of Trajectories Based on Segment Splitting
Source: J Chem Inf Model. 2021 Oct 5;61(10):5066–81. doi: 10.1021/acs.jcim.1c00598 (PMC8549068; doi:10.1021/acs.jcim.1c00598)
Supplement: Supplementary file 1 — ci1c00598_si_001.pdf [file ci1c00598_si_001.pdf]

Supplementary information for:

CATBOSS: Cluster Analysis of Trajectories Based on Segment Splitting

Jovan Damjanovic,[1] James M. Murphy,[2,**] and Yu-Shan Lin[1,*]

[1] Department of Chemistry, Tufts University, Medford, Massachusetts, 02155, United States
[2] Department of Mathematics, Tufts University, Medford, Massachusetts, 02155, United States

Methods

Molecular dynamics simulations. Both model systems simulated by our group (alanine dipeptide and valine dipeptide) were simulated using conventional molecular dynamics (MD). The simulations were conducted in the GROMACS 2018 package,[1] using the RSFF2 force field and the TIP3P water model.[2,3] The RSFF2 force field was parameterized using a coil library and shown to recapitulate the intrinsic preferences of amino acids, as shown on the example of dipeptides.[2]

Each system was simulated twice, starting from different initial conformations, in order to verify simulation convergence. Both initial structures were solvated in a cubic box of pre-equilibrated water, with a minimum distance between the peptide and the walls of the box set to 1.0 nm. The steepest descent algorithm was used to minimize the solvated structure. Upon minimization, the system was equilibrated in two stages: first, 50 ps of *NVT* simulation at 300 K and 50 ps of *NPT* simulation at 300 K and 1 bar were performed with all peptide heavy atoms position-restrained. In the second stage, the position restraints were released, and the same sequence of *NVT* and *NPT* simulations was run again, this time for 100 ps each. Production runs of 200 ns (for alanine dipeptide) and 250 ns (for valine dipeptide) were then performed in the *NPT* ensemble, at 300 K and 1 bar, with a 2 fs time step and data recorded every step.

The simulation was run using the leapfrog algorithm, with water geometry maintained using SETTLE and hydrogen-containing bonds constrained to equilibrium lengths using LINCS. The nonbonded interaction cutoff was set to 1.0 nm, with Coulombic interactions beyond the cutoff computed using particle mesh Ewald summation, with a Fourier spacing of 0.12 nm and cubic interpolation. Dispersion corrections for both energy and pressure were applied to the long-range van der Waals interactions. Temperature was controlled by velocity rescaling, with a coupling time constant of 0.1 ps. The Berendsen barostat was used for pressure control, with a coupling time constant of 2.0 ps and isothermal compressibility of 4.5×10^{-5} bar^{-1}.

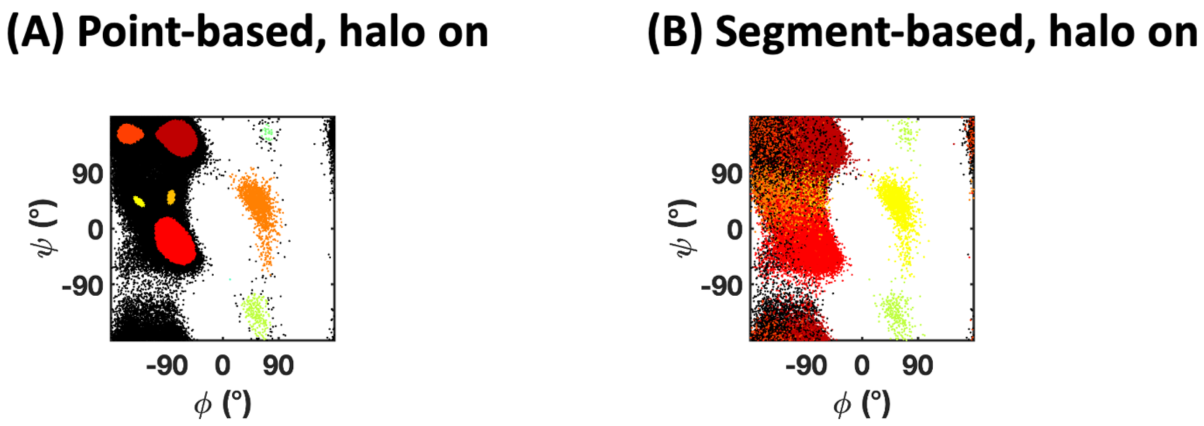

Figure S1. Amount of data discarded (shown in black) when halo control (non-classification of noise points) is applied. **(A)** Point-based density-peak clustering; **(B)** CATBOSS.

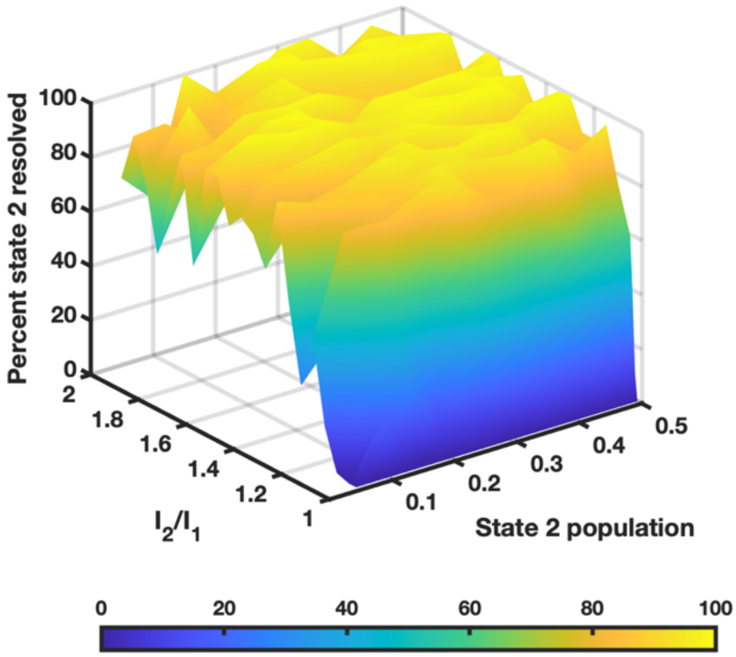

Figure S2. Performance of Li and Yang's clustering algorithm with number of clusters manually set to 2.

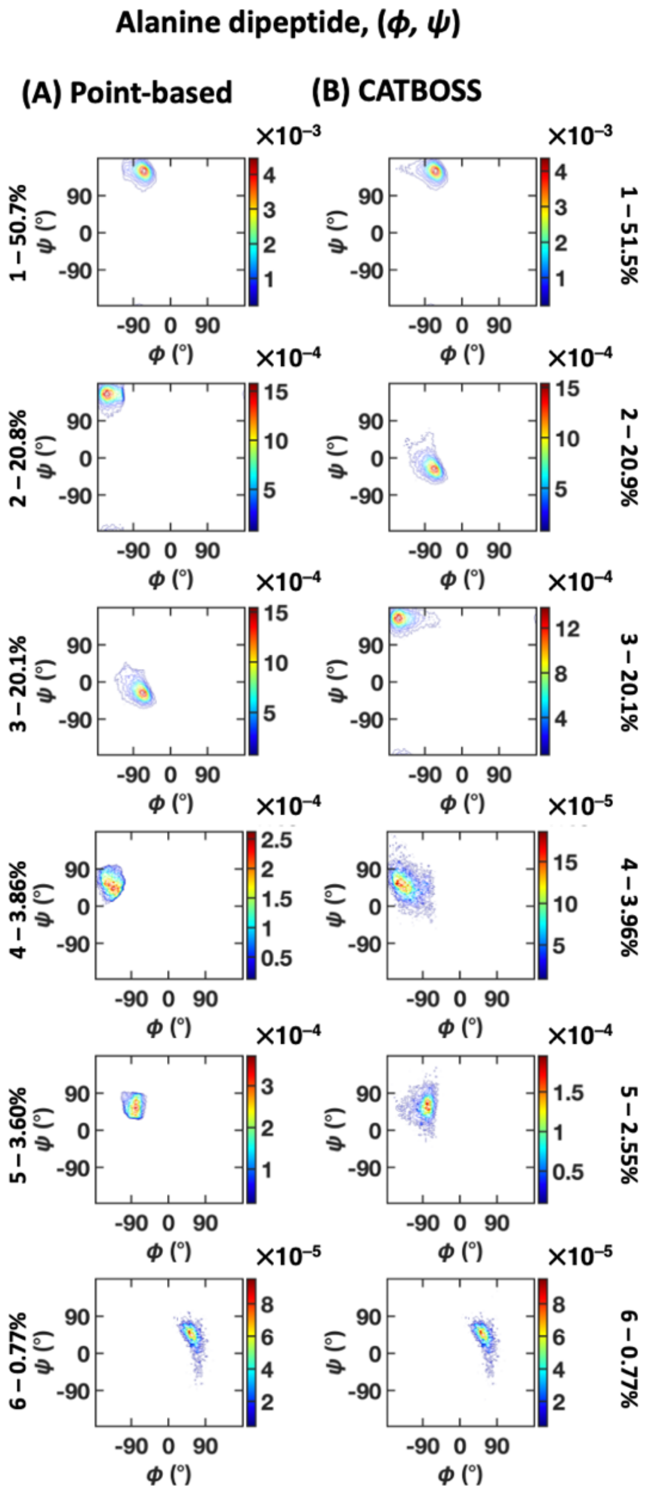

Figure S3. Six top-populated clusters from the alanine dipeptide data set, shown as density contour plots. (**A**) Point-based density-peak clustering; **(B)** CATBOSS.

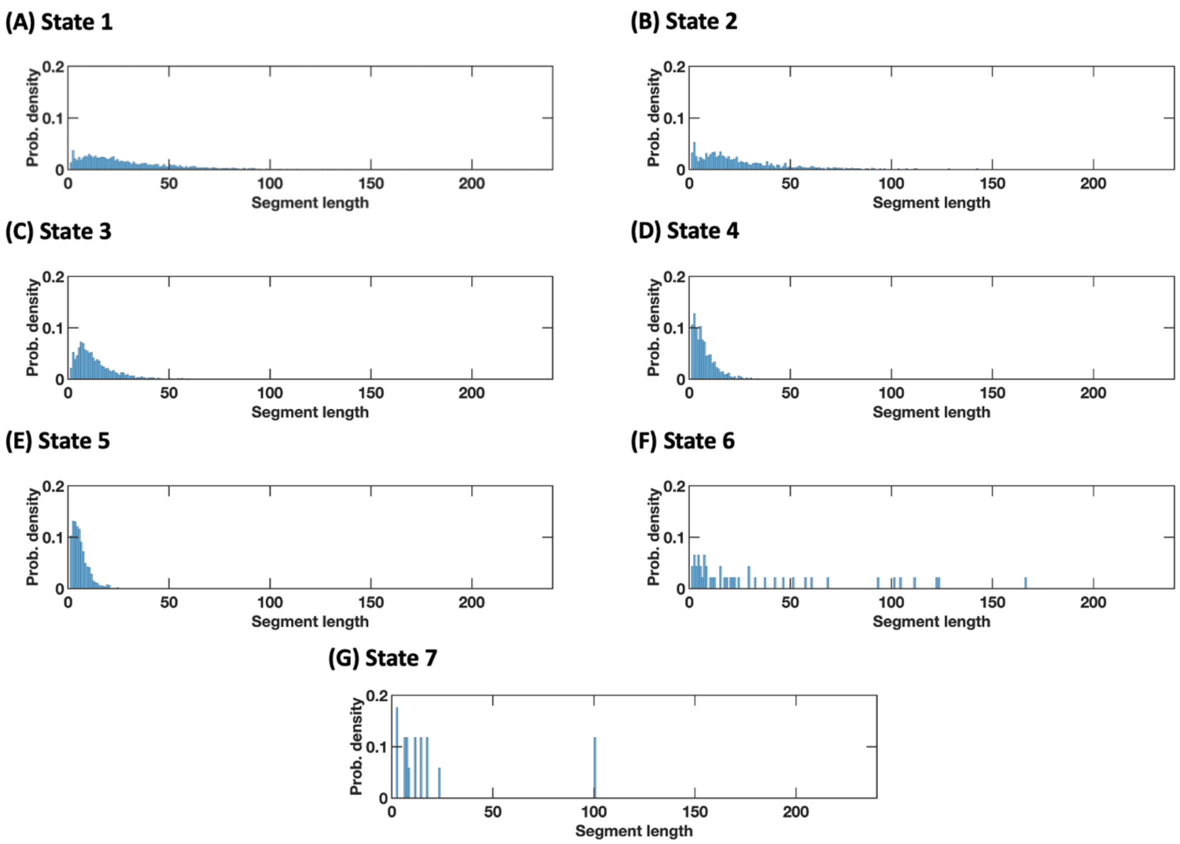

Figure S4. Segment length distributions by state for the alanine dipeptide data set. Note that states 4 and 5 predominantly consist of short segments.

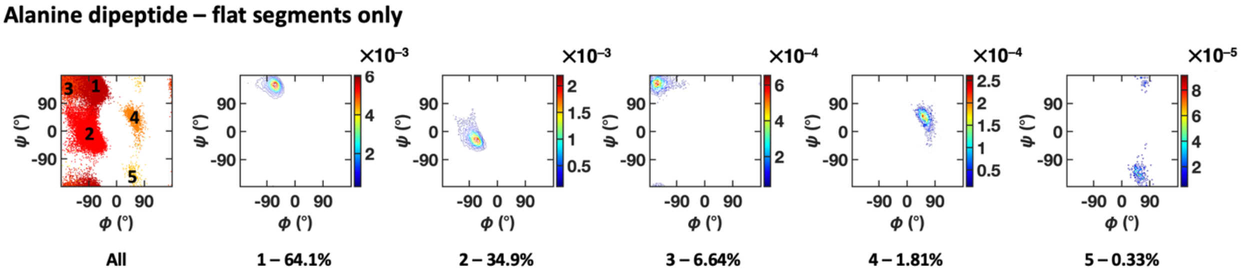

Figure S5. CATBOSS cluster assignments when applied to flat segments of the alanine dipeptide data set only.

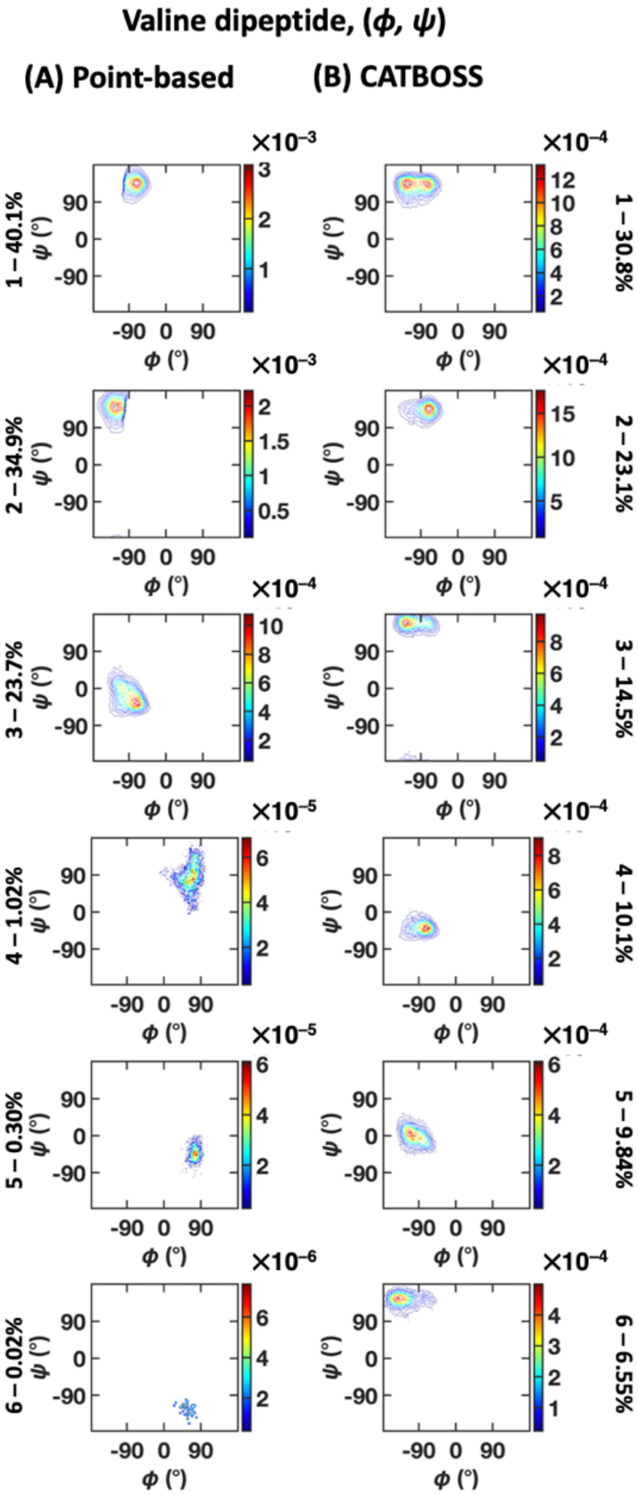

Figure S6. Six top-populated clusters from the two-dimensional valine dipeptide data set, shown as density contour plots. **(A)** Point-based density-peak clustering; **(B)** CATBOSS.

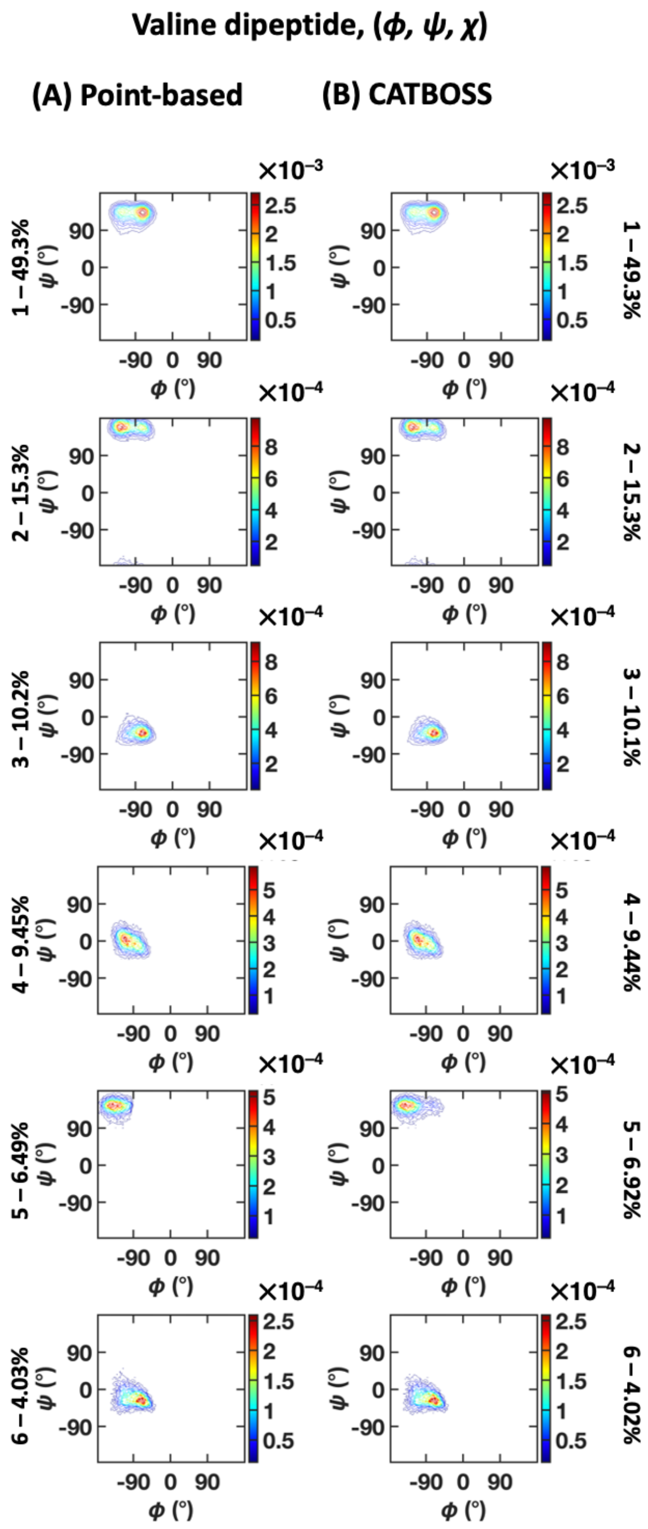

Figure S7. Six top-populated clusters from the three-dimensional valine dipeptide data set, shown as density contour plots. **(A)** Point-based density-peak clustering; **(B)** CATBOSS.

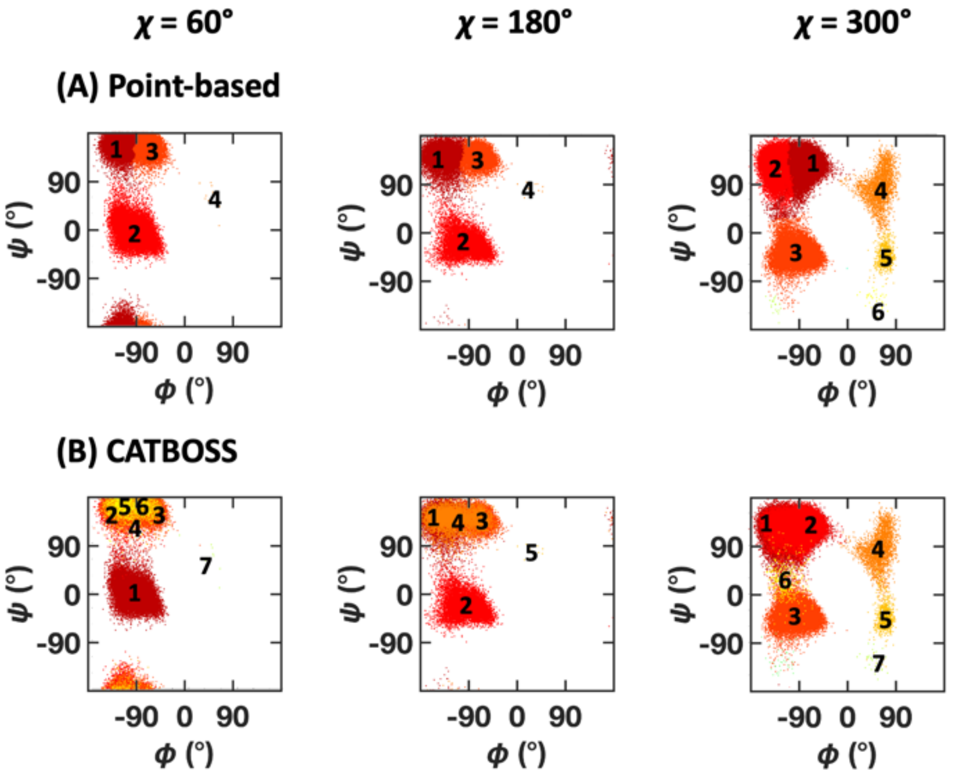

Figure S8. Cluster assignments of separately clustered χ conformers within the three-dimensional valine dipeptide data set. **(A)** Point-based density-peak clustering; **(B)** CATBOSS.

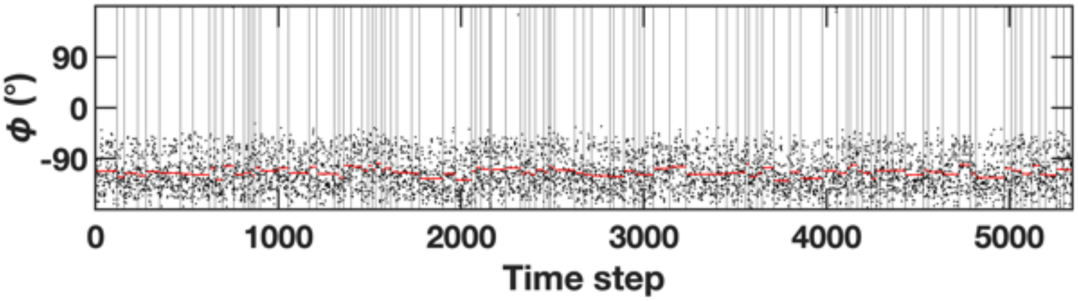

Figure S9. Concatenated view of segments belonging to an artifact cluster in the 180° χ conformer subset of the three-dimensional valine dipeptide data set (**Figure S4**, middle panel, state 4). Computed segment means are indicated in red. Note the apparent bimodality of the segments. Note that only the angle ϕ is shown, as it is the only variable in which the two states these segments traverse significantly differ.

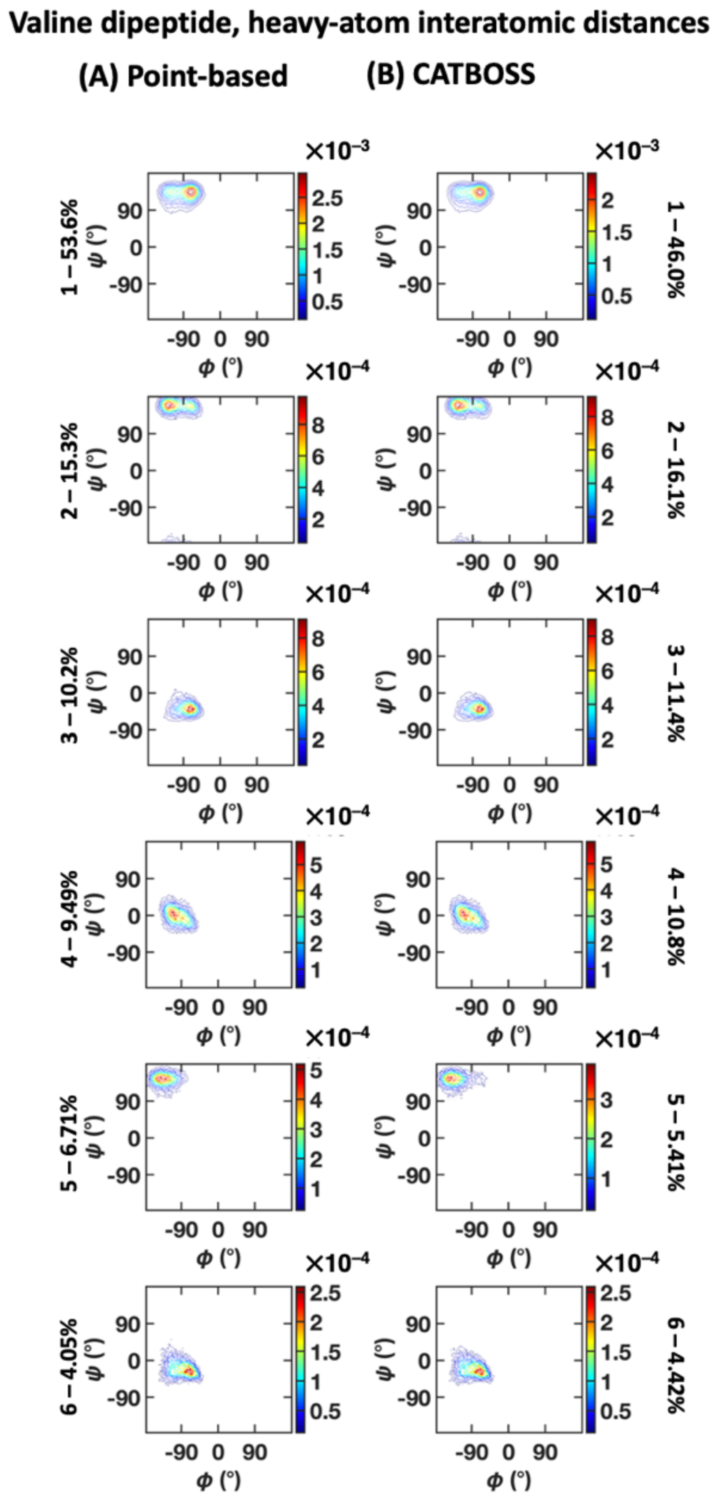

Figure S10. Six top-populated clusters from the valine dipeptide interatomic distances data set, shown as density contour plots. **(A)** Point-based density-peak clustering; **(B)** CATBOSS.

Cluster 1

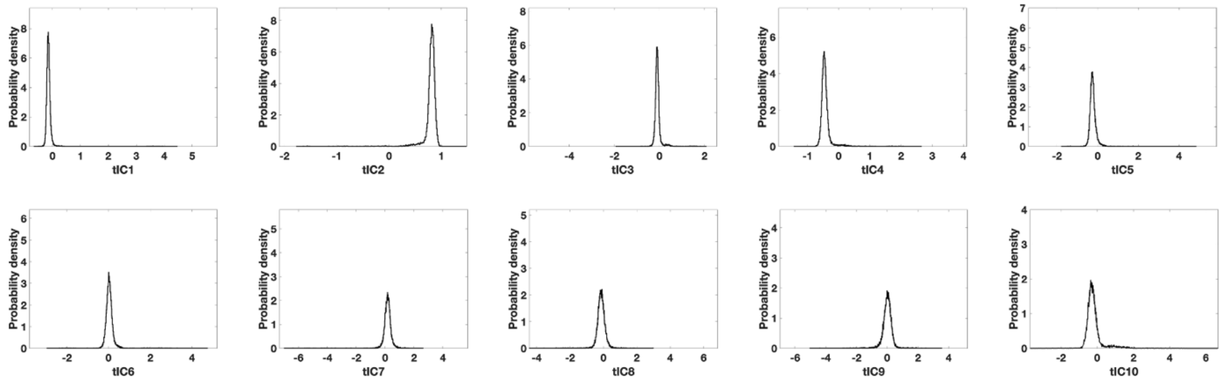

Cluster 2

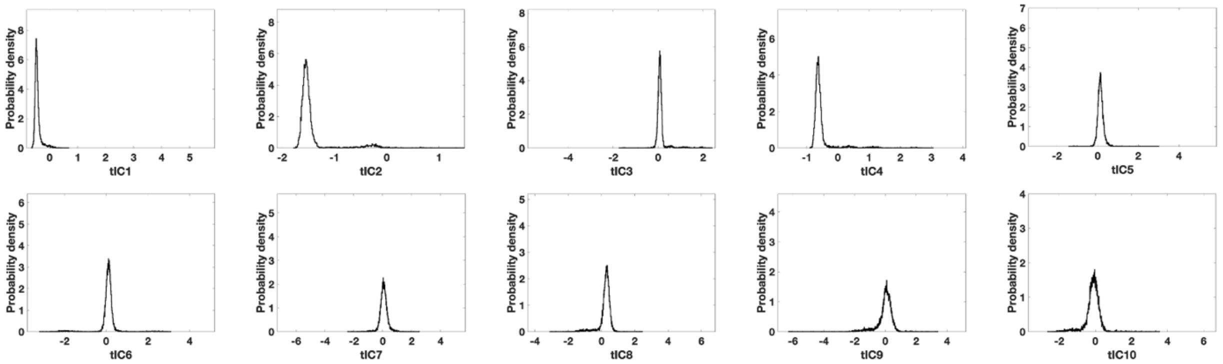

Cluster 3

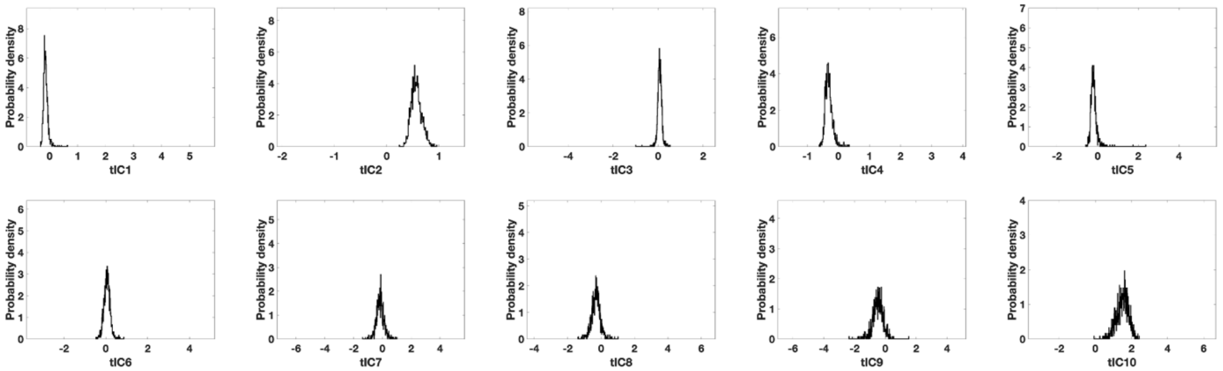

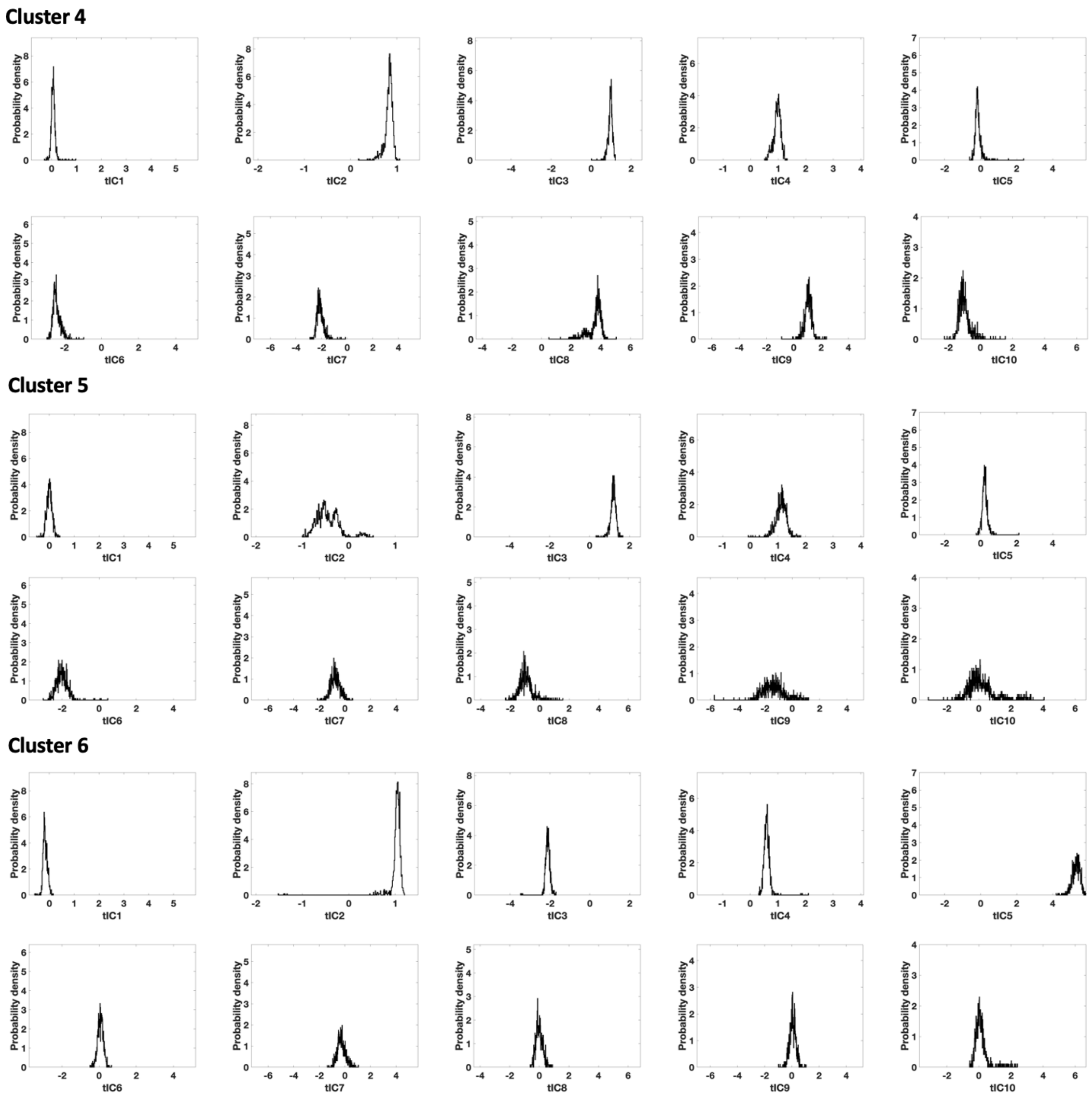

Figure S11. Individual independent component distributions for the top six clusters of the BPTI data set detected by CATBOSS. The sharp, unimodal distributions suggest clean separation and well-defined states.

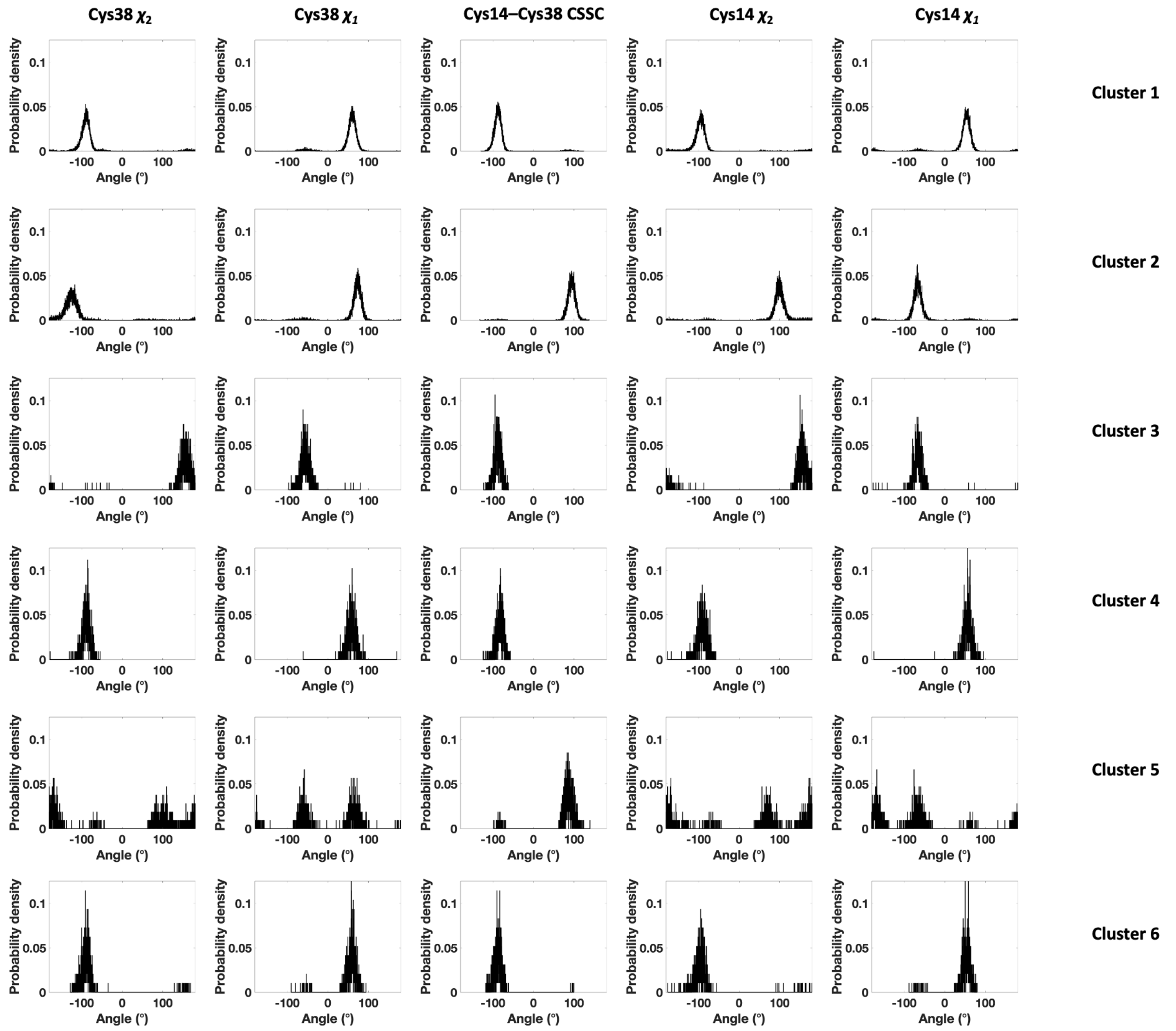

Figure S12. Distributions of the Cys14–Cys38 disulfide bridge dihedral angles for the top six clusters of the BPTI data set detected by CATBOSS. The similarity between clusters 1, 4, and 6 (which differ in tIC distributions) suggests that this disulfide bridge is not the only discriminating factor. Note also cluster 5, in which the two cysteine residues are far apart in space.

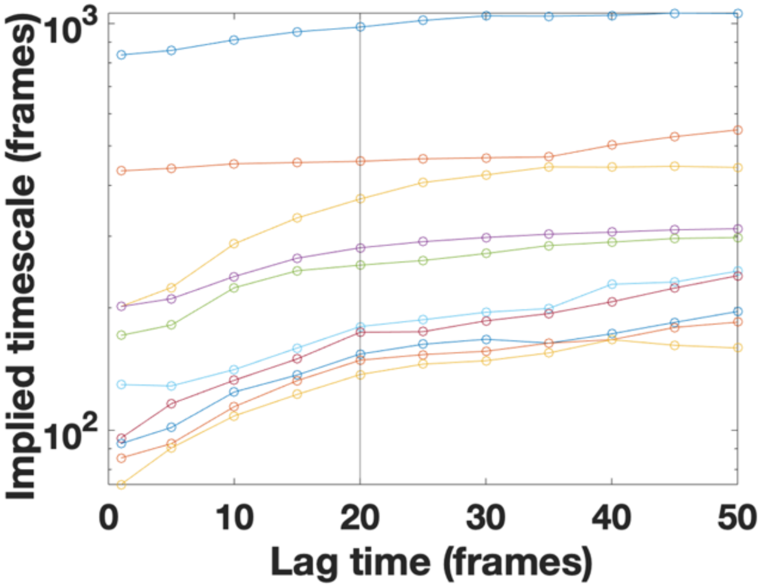

Figure S13. Ten slowest-relaxing implied timescales using CATBOSS-detected clusters of the BPTI trajectory as state input. 1 frame = 25 ns. Note that 20 frames = 500 ns is a reasonable lag time to use.

Cluster 1

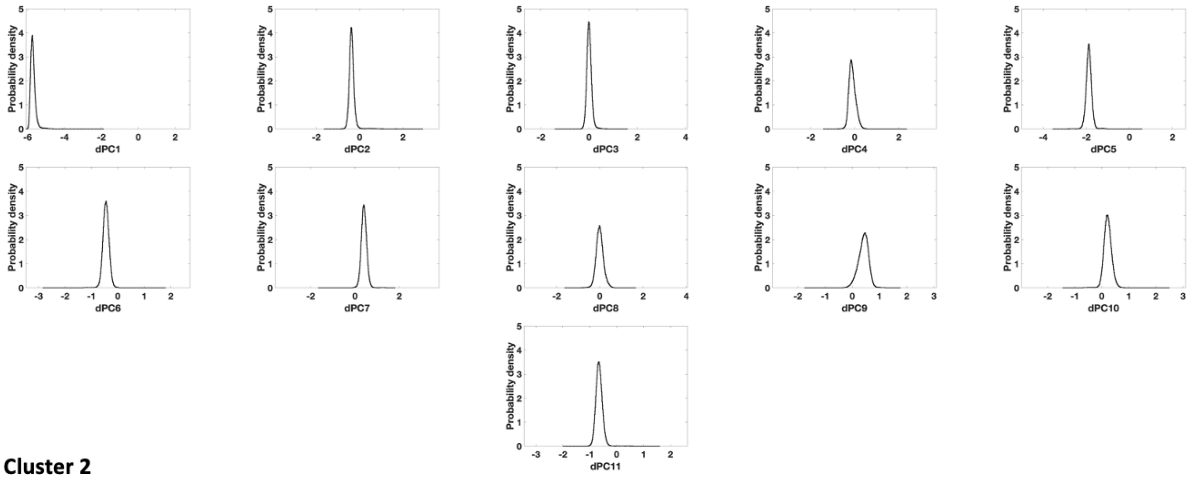

Cluster 2

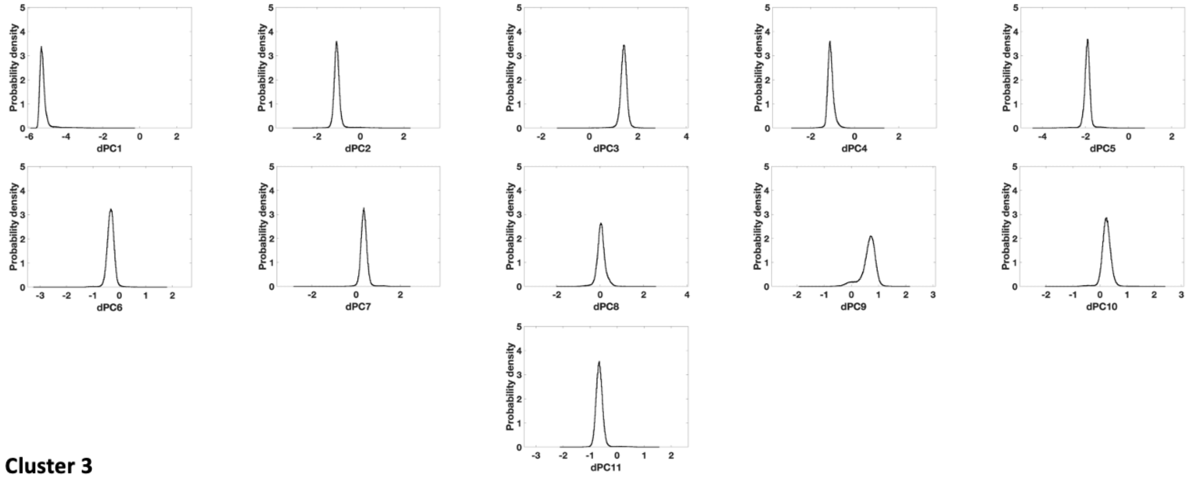

Cluster 3

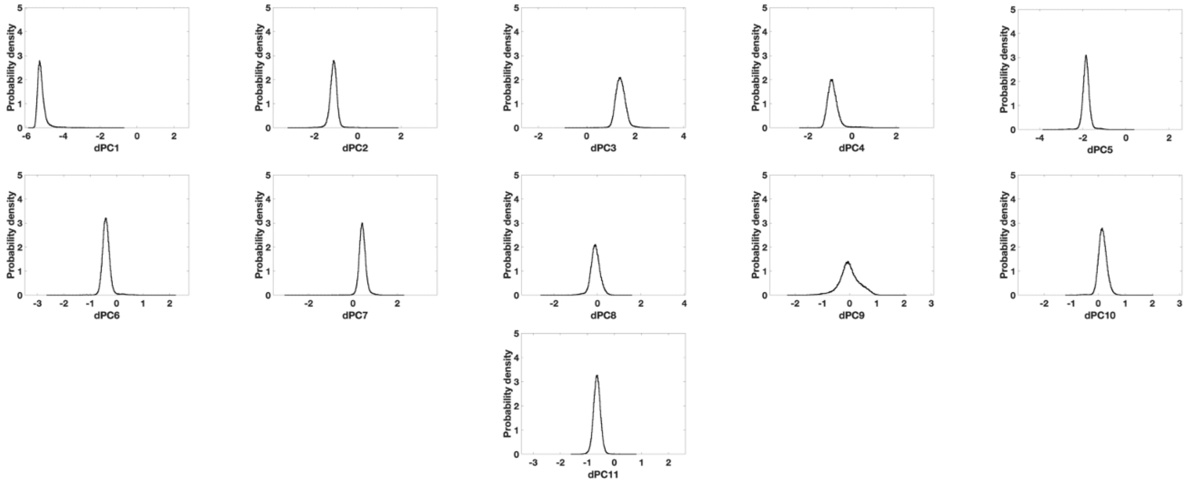

Figure S14. Individual dihedral principal component distributions for the top three clusters of the HP35 data set detected by CATBOSS. The sharp, unimodal distributions suggest clean separation and well-defined states.

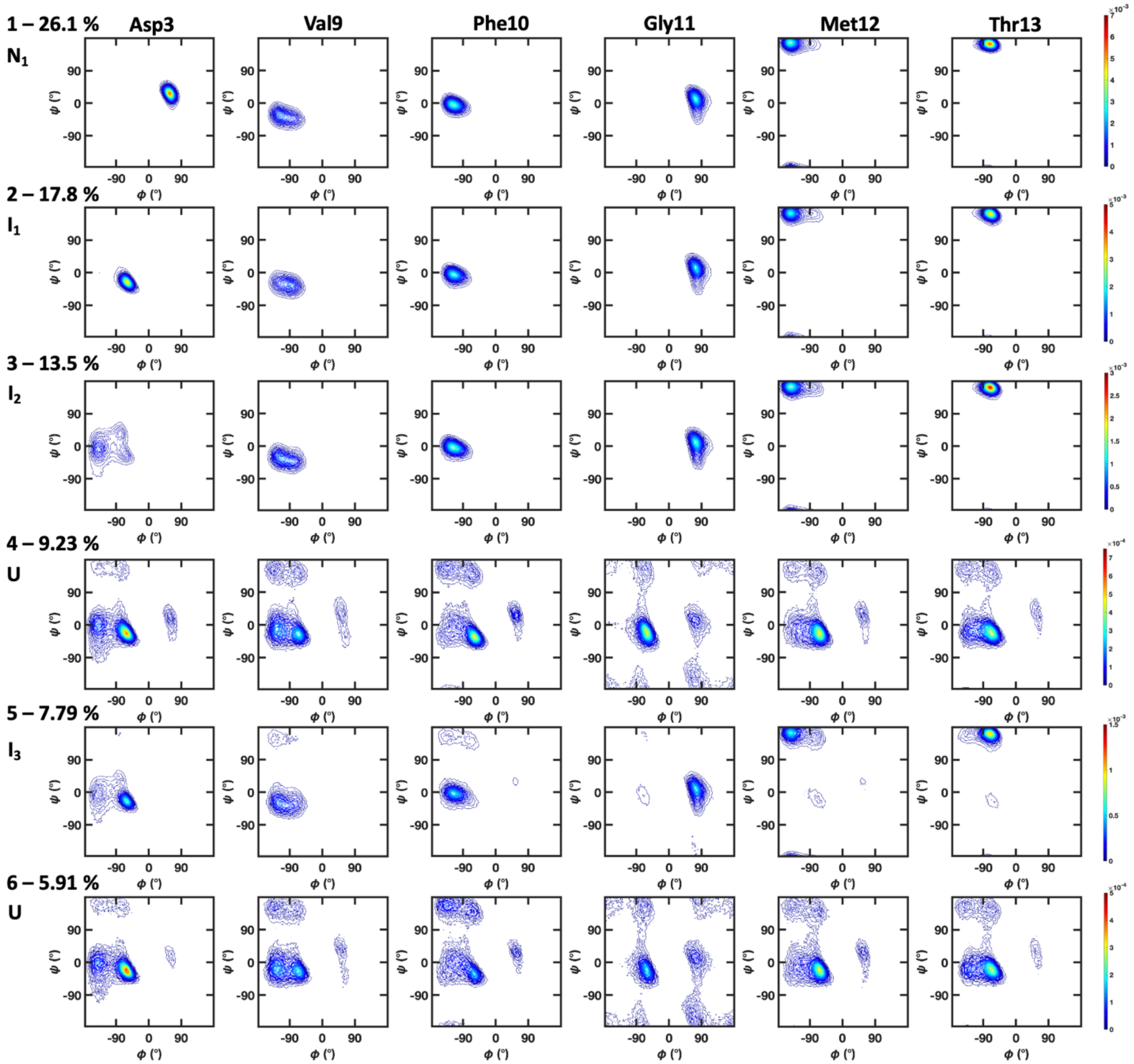

Figure S15. Density contour plots of residues 3, 9–13 for the top six clusters of the HP35 dPC trajectory clustered by CATBOSS. Note the presence of native-like (N), intermediate (I), and unfolded (U) states.

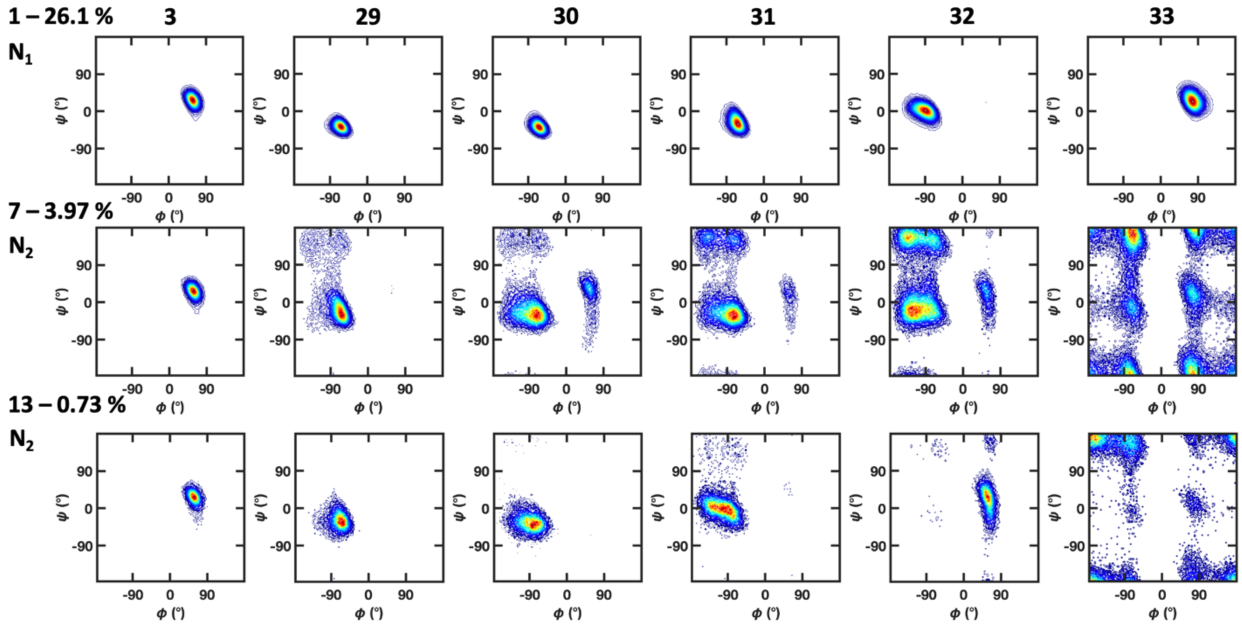

Figure S16. Density contour plots of residues 3, 29–33 for the top three native-like clusters of the HP35 dPC trajectory clustered by CATBOSS. For visual clarity, each plot consists of 100 contours evenly spaced between the minimum and maximum density value for that plot. Note that clusters 7 and 13 would both be classified as N_2 by MPP.

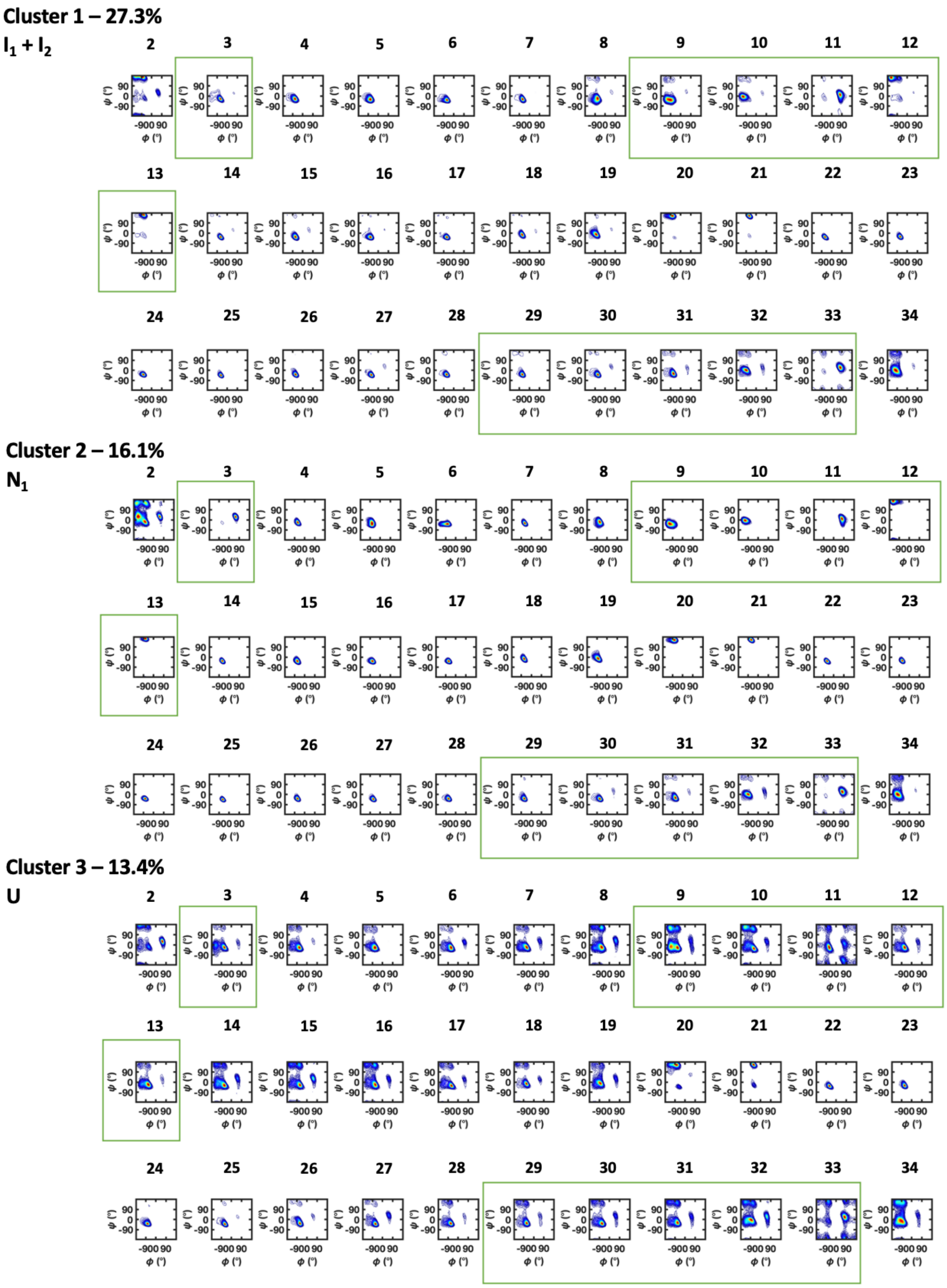

Cluster 1 – 27.3%
$I_1 + I_2$
Cluster 2 – 16.1%
N_1
Cluster 3 – 13.4%
U
ψ (°)
φ (°)

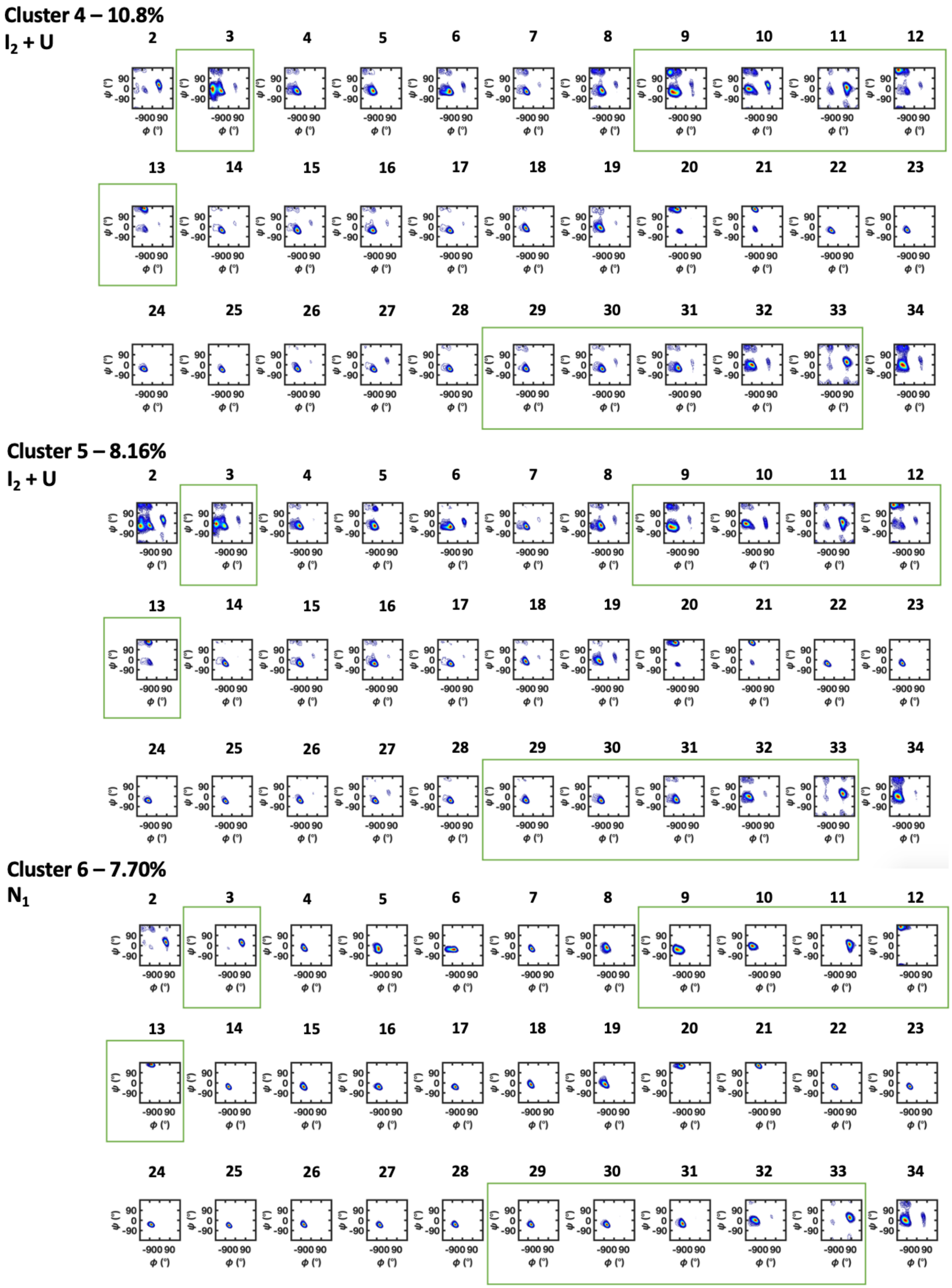

Figure S17. Full backbone dihedral distributions for the top 6 clusters (as determined by CATBOSS) of the raw HP35 backbone dihedral trajectory. For visual clarity, each plot consists of

100 contours evenly spaced between the minimum and maximum density value for that plot. Note the differences in residues in positions 3, 9–13, and 29–33.

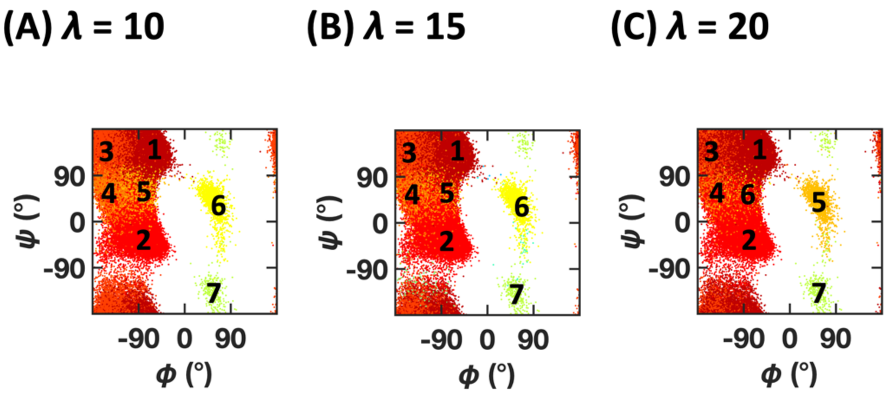

Figure S18. CATBOSS cluster assignment dependence on the tuning parameter λ for the alanine dipeptide data set. **(A)** $\lambda = 10$ (10,415 segments); **(B)** $\lambda = 15$ (6,140 segments); **(C)** $\lambda = 20$ (4,169 segments).

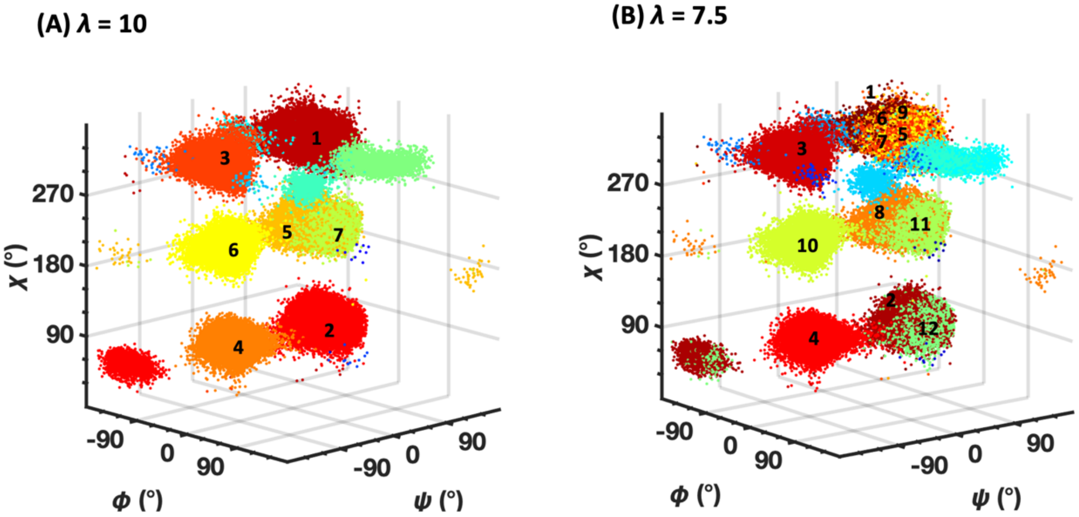

Figure S19. Cluster assignment dependence on the tuning parameter λ for the three-dimensional valine dipeptide data set. **(A)** λ = 10 (5,353 segments); **(B)** λ = 7.5 (14,529 segments). More sensitive change detection yields more separation in this situation.

References

(1) Abraham, M. J.; Murtola, T.; Schulz, R.; Páll, S.; Smith, J. C.; Hess, B.; Lindahl, E. GROMACS: High Performance Molecular Simulations through Multi-Level Parallelism from Laptops to

Supercomputers. *SoftwareX* **2015**, *1–2*, 19–25. https://doi.org/10.1016/j.softx.2015.06.001.
(2) Zhou, C.-Y.; Jiang, F.; Wu, Y.-D. Residue-Specific Force Field Based on Protein Coil Library. RSFF2: Modification of AMBER Ff99SB. *J. Phys. Chem. B* **2015**, *119*, 1035–1047. https://doi.org/10.1021/jp5064676.
(3) Jorgensen, W. L.; Chandrasekhar, J.; Madura, J. D.; Impey, R. W.; Klein, M. L. Comparison of Simple Potential Functions for Simulating Liquid Water. *J. Chem. Phys.* **1983**, *79*, 926–935. https://doi.org/10.1063/1.445869.
